# New Urea Derivatives Are Effective Anti-senescence Compounds Acting Most Likely via a Cytokinin-Independent Mechanism

**DOI:** 10.3389/fpls.2018.01225

**Published:** 2018-09-11

**Authors:** Jaroslav Nisler, Marek Zatloukal, Roman Sobotka, Jan Pilný, Barbora Zdvihalová, Ondrej Novák, Miroslav Strnad, Lukáš Spíchal

**Affiliations:** ^1^Laboratory of Growth Regulators, Centre of the Region Haná for Biotechnological and Agricultural Research, Institute of Experimental Botany AS CR & Palacký University, Olomouc, Czechia; ^2^Department of Chemical Biology and Genetics, Centre of the Region Haná for Biotechnological and Agricultural Research, Palacký University, Olomouc, Czechia; ^3^Laboratory of Photosynthesis, Centre Algatech, Institute of Microbiology, Třeboň, Czechia; ^4^Faculty of Science, University of South Bohemia, České Budějovice, Czechia

**Keywords:** ASES, cytokinin, photosystem II, senescence, stress, thidiazuron, wheat

## Abstract

Stress-induced senescence is a global agro-economic problem. Cytokinins are considered to be key plant anti-senescence hormones, but despite this practical function their use in agriculture is limited because cytokinins also inhibit root growth and development. We explored new cytokinin analogs by synthesizing a series of 1,2,3-thiadiazol-5-yl urea derivatives. The most potent compound, 1-(2-methoxy-ethyl)-3-1,2,3-thiadiazol-5-yl urea (ASES - Anti-Senescence Substance), strongly inhibited dark-induced senescence in leaves of wheat (*Triticum aestivum* L.) and *Arabidopsis thaliana*. The inhibitory effect of ASES on wheat leaf senescence was, to the best of our knowledge, the strongest of any known natural or synthetic compound. *In vivo*, ASES also improved the salt tolerance of young wheat plants. Interestingly, ASES did not affect root development of wheat and Arabidopsis, and molecular and classical cytokinin bioassays demonstrated that ASES exhibits very low cytokinin activity. A proteomic analysis of the ASES-treated leaves further revealed that the senescence-induced degradation of photosystem II had been very effectively blocked. Taken together, our results including data from cytokinin content analysis demonstrate that ASES delays leaf senescence by mechanism(s) different from those of cytokinins and, more effectively. No such substance has yet been described in the literature, which makes ASES an interesting tool for research of photosynthesis regulation. Its simple synthesis and high efficiency predetermine ASES to become also a potent plant stress protectant in biotechnology and agricultural industries.

## Introduction

In general, there are two types of leaf senescence-developmental (natural) leaf senescence and stress-induced leaf senescence (e.g., Becker and Apel, 1993; Oh et al., 1996). Developmental leaf senescence is an age-dependent process and precedes leaf death (e.g., Hensel et al., 1993; Jing et al., 2005), while the other type is induced by an external stimulus. Stress-induced plant senescence leads to a premature aging of plant organs, damages them and leads to losses in crop yields. For this reason, the discovery of technologies that can retard plant senescence has become highly desirable.

From the plethora of the literature available it is apparent that different stressors induce senescence through different signaling molecules/phytohormones and pathways. For example drought, salinity and low temperatures were shown to induce senescence in Arabidopsis through the elevation of abscisic acid (Yang et al., 2011). Shadow or complete darkness triggers senescence through an ethylene-response pathway (Buchanan-Wollaston et al., 2005) and, a decline in photosynthetic activity which leads to reduced sugar level also induces senescence (Hensel et al., 1993; Quirino et al., 2000; Liebsch and Keech, 2016). Ethylene production also accompanies and accelerates the senescence of wounded or detached leaves (Abeles et al., 1988; Ferrante et al., 2002). For comparison, it was shown that salicylic acid response pathway plays a major role in the developmental senescence of Arabidopsis leaves (Buchanan-Wollaston et al., 2005).

Although it is clear that different senescence-promoting signals use different signaling pathways, it has been shown that these multiple pathways are interconnected to form a regulatory network which controls and execute the senescence (He et al., 2001; Gan, 2003). For example, it was shown that developmental and salt-triggered leaf senescence shares the same H_2_O_2_ signaling pathways (Allu et al., 2014). This conclusion was based on an extensive overlap found between the transcriptome of both types of senescence in Arabidopsis. The modern multi-omics approaches indicate that the senescence process itself exhibits a certain pattern at the cellular and molecular levels regardless of the origin of its initial promoting factor (Gan and Amasino, 1997; Gan, 2003; Guo and Gan, 2012; reviewed in Kim et al., 2016). Using the rice coleoptile it was shown that the degradation of chloroplast DNA occurred first, followed by the degradation of the chloroplast inner membranes and of ribulose-1,5-bisphosphate carboxylase/oxygenase (a chloroplast-targeted photosynthetic enzyme) and simultaneous condensation and disorganization of the nucleus. Finally, the cell wall collapsed and all cellular components were lost (Inada et al., 1998). It is expected that chloroplasts are broken down first, because they contain the majority of the nitrogen in a leaf, while the nucleus remains intact to control the recycling process (Makino and Osmond, 1991). Macroscopically, the leaf yellowing is the first visible sign of leaf senescence and results from the preferential degradation of the chlorophyll but not the carotenoids, which are yellow-red pigments (reviewed by Matile et al., 1999). In *Arabidopsis thaliana*, the visible yellowing as well as the chlorophyll loss are widely used to stage the progression of senescence, which reproducibly correlate with other biochemical changes that occur during leaf senescence (e.g., Hensel et al., 1993; Lohman et al., 1994; Jing et al., 2002).

It is well known that substances such as abscisic acid, ethylene, jasmonic acid, and brassinosteroids promote plant senescence (reviewed e.g., in Ghorbani-Javid et al., 2011; Gepstein and Glick, 2013), while polyamines (Wei-yu et al., 1990), gibberellins (Jordi et al., 1995), and cytokinins (e.g., Richmond and Lang, 1957; Gan and Amasino, 1995; Ferrante et al., 2002), postpones plant senescence. Until now, the best effects had been achieved through the application of cytokinins (Gan and Amasino, 1995; Ferrante et al., 2002; Rivero et al., 2007), which are considered to be the key phytohormones that retard leaf senescence and enhance plant stress tolerance (Gan, 2003). Excellent examples can be found from research by Gan and Amasino (1995) and Rivero et al. (2007), in which the endogenous levels of cytokinins were enhanced during the onset of senescence in transgenic tobacco plants. These researchers used senescence-, maturation-, and drought stress-associated promoters to control the expression of the gene encoding isopentenyl transferase, which is a key enzyme in cytokinin biosynthesis (EC 2.5.1.27; Ihara et al., 1984). Enhanced levels of cytokinins retarded leaf senescence and prolonged the photosynthetically-active life-span of both leaves and plants. Delayed leaf senescence was also shown to induce extreme drought tolerance in *Nicotiana tabacum* (Rivero et al., 2007) and several other reports revealed the role of cytokinins during drought, cold, salt and other stresses (e.g., Tran et al., 2007; Jeon et al., 2010; Nishiyama et al., 2011). Several mechanisms through which cytokinins delay plant senescence have been proposed. It is known that cytokinins are involved in the control of chloroplast biogenesis and degradation (Jordi et al., 2000), as well as pigment accumulation and mediation of the activities of chloroplast enzymes (Lerbs et al., 1984; Kusnetsov et al., 1994; Yaronskaya et al., 2006). It has been shown that higher cytokinin content induced an antioxidant protection mechanism in chloroplasts of *Nicotiana tabacum* during leaf senescence (Procházková et al., 2008). One recent research further demonstrated that cytokinins are implemented in the regular repair of D1 protein, which is necessary for the functioning of photosystem II (PSII). The analysis of cytokinin receptor mutants revealed that the protective function of the cytokinin during light stress depends on the ARABIDOPSIS HISTIDINE KINASE2 (AHK2) and AHK3 receptors and the type B ARABIDOPSIS RESPONSE REGULATOR1 (ARR1) and ARR12 (Cortleven et al., 2014). Previous research has also shown that AHK3, one of the three cytokinin receptors in *Arabidopsis thaliana*, plays a crucial role in cytokinin-mediated leaf longevity through the specific phosphorylation of cytokinin response regulator ARR2 (Kim et al., 2006). It is also known that cytokinins inhibit the transcription of most of the Senescence Associated Genes (SAG, Weaver et al., 1998). SAGs are genes which are expressed during senescence and encode degradative enzymes, such as RNAses, proteinases and lipases, and products involved in nutrient translocation processes (reviewed in Gan and Amasino, 1997). Beside this, cytokinins can also upregulate the expression of extracellular cell wall invertase, an enzyme which has a crucial role in source-sink regulation (Balibrea Lara et al., 2004).

In addition to their involvement in plant senescence, cytokinins have many other essential roles in plant growth and development (reviewed by Mok, 1994). They are, inter alia, positive regulators of shoot growth but negative regulators of root development (e.g., Werner et al., 2003). In Arabidopsis, it was also shown that cytokinin signaling negatively affects the seed germination (Riefler et al., 2006). For these reasons, cytokinins are rarely applied to seeds or young plantlets, which do not have properly developed roots yet and therefore, these hormones are only utilized to a minor extent in horticulture and agriculture. On the other hand, substances that could delay plant senescence and death under different stress conditions are very desirable for the agricultural and biotechnology industries.

Other types of anti-senescence compounds include gibberellins or polyamines; however these substances have much lower anti-senescence activity than cytokinins (Wei-yu et al., 1990; Ferrante et al., 2002). Furthermore, the role of gibberellins in senescence is not universal but rather species-specific (Fletcher and Osborne, 1965). For instance, a greater sensitivity to drought stress was observed in Arabidopsis mutants with elevated levels of gibberellins, and *vice versa* (Colebrook et al., 2014). Only a few synthetic compounds without cytokinin-like structure which delay leaf senescence have been described. However, these compounds most probably also function *via* enhancing cytokinin levels in plants. The compounds were discovered among fungicides and include triazoles and strobilurins. As a side effect of both compound groups it was found that they cause accumulation of cytokinins (Fletcher and Arnold, 1986; Grossmann and Retzlaff, 1997) which leads to the delayed leaf senescence in wheat, peas and soybeans (Fletcher and Nath, 1984; Fletcher and Hofsta, 1985) and increases stress tolerance of wheat toward drought and heat (Wu and von, 2002; Jaleel et al., 2006). In conclusion, the substances known to be potent inhibitors of leaf senescence are cytokinins or compounds increasing their content in plants. Both groups of compounds delay senescence by the mechanisms described herein for cytokinins. So far, no substances that would be more effective than cytokinins in delaying plant senescence have been described.

Here we designed and synthesized a spectrum of 1,2,3-thiadiazol-5-yl urea derivatives and tested them for anti-senescence activity. Because this type of biological activity is primarily exhibited by cytokinins, all our derivatives were also screened for cytokinin activity in other cytokinin bioassays. The activities of all compounds were compared to those of thidiazuron (TDZ), which currently seems to be the strongest cytokinin (Mok et al., 1982; Thomas and Katterman, 1986; Spíchal et al., 2004). Based on our results, we identified and characterized novel, extremely potent inhibitors of leaf senescence whose mode of action is different from the mechanisms that have previously been described for cytokinins. Further, we demonstrate that ASES, unlike TDZ, inhibits the stress-induced degradation of PSII in detached wheat leaves.

## Materials and methods

### Chemicals

1,2,3-Thiadiazol-5-ylamine was supplied by TCI Europe (Zwijndrecht, Belgium). TDZ, *trans-*zeatin (*t*Z), N^6^-benzyladenine (BA), (*E*)-4-hydroxy-3-methyl-but-2-enyl amine and 3-methyl-but-2-enyl amine were supplied by OlchemIm (Olomouc, Czech Republic). All other chemicals were purchased from Sigma Aldrich (Munich, Germany).

### General synthetic procedures

The compound 1,2,3-thiadiazol-5-ylamine was the starting material for the synthesis of 5-isocyanato-1,2,3-thiadiazole, which was prepared as described by Nisler et al. (2016). The compounds TD-*t*Z, TD-iP, TD-DHZ and compounds 1-15 were prepared by mixing 5-isocyanato-1,2,3-thiadiazole and the corresponding commercially available amine under mild heat (30 to 60°C) in tetrahydrofuran in the presence of a catalytic amount of triethylamine (Goldschmidt and Bardach, 1892). The conversion process was monitored by thin layer chromatography. Since aliphatic amines are not visible under ultraviolet light, the staining with ninhydrine was used to visualize the consumption of an amine. The reaction lasted from 1 h to the maximum of 12 h. After the reaction, the solvent was evaporated, resulting in a solid/semisolid residue that was then purified by flash silica column chromatography using 63 micron Chromatographic silica (Davisil) and CHCl_3_:MeOH (9:1) mobile phase. The compounds were usually prepared in milligram quantities and the yields varied between 30 and 80%. When the starting amine contained a free hydroxyl group, it was O-protected with a *tert*-butyldimethylsilyl group prior to condensation with 5-isocyanato-1,2,3-thiadiazole and then de-protected with hydrochloric acid (Greene and Wuts, 1991).

### General experimental procedures

Analytical thin-layer chromatography was carried out using silica gel 60 WF_254_ plates (Merck). The purity of the synthesized compounds was confirmed by high performance liquid chromatography (HPLC, Beckman Gold System) with diode array detection (Beckman Gold System, California), and was always above 97%. The elemental composition of the prepared compounds was confirmed by HPLC-(ESI+)-MS QqTOF (Q-Tof TOF micro™ Waters MS Technologies, Milford, MA). For details see Supplementary Table S1. ^1^H NMR spectra were measured on a Jeol 500-SS spectrometer (Jeol, Peabody, MA) operating at a frequency of 500.13 MHz (Supplementary Table S2).

### Cytokinin bioassays

Tobacco callus, Amaranthus and Wheat leaf senescence assay (WLSA) were performed as described by Holub et al. (1998) including the number of repetitions in each assay. An exception to the published protocols was that the tobacco callus assay was performed in 6-well microtiter plates (with each well containing 3 ml of Murashige Skoog medium into which 0.1 g of callus was added). Each compound was tested at least twice in each assay and in a wider concentration range to obtain concentration-dependent activity curves. These curves were evaluated using Scion image program to obtain the IC_50_ and EC_50_ values for each compound (presented in Table 2). IC_50_ means compound concentration at which chlorophyll degradation is inhibited by 50% and EC_50_ means half the maximal effective concentration.

Amaranthus assay evaluates the induction of betacyanin synthesis in *Amaranthus* (*Amaranthus caudatus var. atropurpurea*) cotyledons, which is induced by cytokinins (Bamberger and Mayer, 1960). The WLSA evaluates the ability of compounds to retard chlorophyll degradation in excised wheat leaves that are kept in the dark. The wheat leaf segments (*Triticum aestivum* cv. Hereward) were used in all other experiments requiring wheat. Arabidopsis (*Arabidopsis thaliana*) leaf senescence assay was performed with detached leaves from 30-day-old plants, which were grown in soil in phytotron under standard conditions (Rivero et al., 2014). Four leaves of a combined weight of 60 mg were placed in a six-well plate and stored in the dark for 12 days at 22°C. The leaves (except the fresh control leaves) floated on water solutions of tested compounds. Fresh control leaves were leaves detached from a plant and immediately used for chlorophyll determination. The duration of the senescence assay of Arabidopsis leaves was prolonged to highlight the difference in chlorophyll content between a negative control and treatments. Arabidopsis leaves were losing chlorophyll more slowly than wheat leaves. Chlorophyll extraction and spectrophotometric determination of its content was done according to Holub et al. (1998).

### Cytokinin receptor activation assay

*Escherichia coli* strain KMI001, harboring either the plasmid pIN-IIIAHK4 or pSTV28-AHK3, which express functional forms of the Arabidopsis cytokinin receptors - histidine kinases CRE1 (Cytokinin Response 1)/AHK4 or AHK3, respectively (Suzuki et al., 2001; Yamada et al., 2001), was used in the experiments. Bacterial strains were kindly provided by T. Mizuno (Japan) and the assay was performed as previously described (Spíchal et al., 2004). Briefly, if a compound is able to activate the specific cytokinin receptor, which is located in a bacterial plasma membrane, bacterial signal transduction system activates the transcription of *LacZ* gene (gene for β-galactosidase). The activity of this enzyme is finally measured in the assay and corresponds to the ability of the tested compound to activate this cytokinin receptor. Both assays were done at least twice and the presented graphs are the most representative examples.

### Arabidopsis *ARR5:GUS* reporter gene assay

Transgenic Arabidopsis plants (*Arabidopsis thaliana* (L.) Heynh. accession Wassilewskija) harboring the *GUS* reporter gene (gene for β-glucuronidase) fused to 1.6 kb of the *ARR5* (*P*_*ARR*5−_*GUS*) gene promoter (D'Agostino et al., 2000) were kindly provided by professor Thomas Schmülling. The assay was performed according to Romanov et al. (2002). β-Glucuronidase (GUS) activity was quantitatively determined by measuring fluorescence of 4-methylumbelliferyl (MU) on a Synergy H4 hybrid reader (Biotek, Winooski, VT) with excitation and emission wavelengths of 365 and 445 nm, respectively. GUS specific activity was determined as nmol MU × mg protein^−1^ × h^−1^. This was calculated by dividing the relative fluorescence units (obtained per hour) by protein content of the same sample. Protein extraction was done as described by Romanov et al. (2002) but its content was determined using a bicinchoninic acid reagent (Smith et al., 1985). Finally we expressed the GUS activity in samples as a relative percentage, the activity of 1 μM BA being set as 100% activation. The concentration-dependent activity curves and EC_50_ values (for TDZ, *t*Z, BA, TD-iP, and TD-*t*Z) were constructed using Scion image program.

### Determination of *Arabidopsis thaliana* cytokinin oxidase/dehydrogenase 2 (AtCKX2) activity

The method based on the coupled redox reaction of phenazine methosulfate and 3-(4,5-dimethylthiazol-2-yl)-2,5-diphenyltetrazolium bromide resulting in the formation of a formazan dye was used to test the ability of the synthesized compounds to inhibit AtCKX2. The assay was performed as described previously (Frébort et al., 2002). Cell-free growth medium of *Saccharomyces cerevisiae* strain 23344c ura- harboring the plasmid pYES2- AtCKX2 was used directly as a source of AtCKX2 (Frébortová et al., 2007).

### Cytokinin analysis

The analysis of endogenous cytokinins, extraction and purification was performed according to the method described by Dobrev and Kamínek (2002) with minor modifications. Briefly, samples (15 mg FW) were homogenized and extracted in 1 ml of modified Bieleski buffer (60% MeOH, 10% HCOOH and 30% H_2_O) together with a cocktail of stable isotope-labeled internal standards (0.25 pmol of cytokinin bases, ribosides, N-glucosides, and 0.5 pmol of cytokinin O-glucosides, nucleotides per sample added). The extracts were purified using the Oasis MCX column (30 mg/1 ml, Waters) conditioned with 1 ml each of 100% MeOH and H_2_O, equilibrated sequentially with 1 ml of 50% (v/v) nitric acid, 1 ml of H_2_O, and 1 ml of 1M HCOOH. After sample application onto an MCX column, un-retained compounds were removed by a wash step using 1 ml of 1M HCOOH and 1 ml 100% MeOH, pre-concentrated analytes were eluted by two-step elution using 1 ml of 0.35M NH4OH aqueous solution and 2 ml of 0.35M NH4OH in 60% (v/v) MeOH solution. The levels of cytokinins were quantified by ultra-high performance liquid chromatography–electrospray tandem mass spectrometry (UHPLC-MS/MS) using stable isotope-labeled internal standards as described by Novák et al. (2008).

### Root elongation assays

Seeds of *Triticum aestivum* were surface sterilized by washing with 96% ethanol (10 min) and sown in a hydroponic growing system (Araponics, Liege, Belgium) using Hoagland's solution supplemented with either 0.1 μM TDZ, 0.1 μM ASES or 0.01% DMSO (solvent control). The plants were grown in a growth chamber at 25°C with a 16/8 h light period at 130 μM m^−2^s^−1^. The root lengths of at least 50 plants were measured 2 weeks after the plants had been sown. Seeds of *Arabidopsis thaliana* (wild-type Col-0) were surface sterilized by the same procedure as seeds of wheat and sown on vertical plates using half-strength Murashige-Skoog medium (supplemented with 0.1% sucrose and 6 g/L phytagel) containing either 0.1 μM TDZ, 0.1 μM ASES or 0.01% DMSO (solvent control). After vernalization (4 days in the dark, 4°C), the plates were transferred into a growth chamber (22°C, 16/8 light/dark, 130 μM m^−2^s^−1^). Root elongation was scored 2 weeks after the transfer, with measurements from at least 40 plants for each treatment. The whole experiment was repeated twice.

### Determination of thiobarbituric acid reactive substances (TBARS) content

Four leaf segments of combined weight of 100 mg from WLSA (5 days in the dark) were used for the determination of TBARS. Each sample was done in five repetitions (5 × 100 mg). The peroxidation of leaf membrane lipids was determined as described by Kováčik et al. (2006), with minor modifications—wheat leaves were extracted with 80% methanol and the trichloroacetic acid content was decreased to 0.1%.

### Ethylene accumulation assay and determination of ethylene production

Two detached leaf tips (final weight 50 mg) from 7-day-old wheat plants were incubated in 10 mL gas chromatography vials with either 1 mL of 0.1% DMSO (control), 10 μM ASES or 10 μM TDZ, and kept in the dark for 6 days at 25°C (1 day longer than the classical WLSA). On the third day, 1 mL of air from each vial was collected and the vials were then left open for 1 h and aerated with fresh air. Another 1 mL of air was collected from each vial on the sixth day. The amount of ethylene in each sample was determined using gas chromatography and the flame ionization detector method described by Fišerová et al. (2001).

### Salt stress experiment

A plastic container was filled with 45 g of perlite and 200 mL of Hoagland's solution (control) or with the addition of ASES to a final concentration of 100 nM. Sterilized seeds of *Triticum a*. cv. Hereward (6 g) were sown on the surface. Both treatments were done in two repetitions. The plants were left to grow in optimal conditions (22°C, 16/8, light/dark, 130 μM m^−2^ s^−1^, humidity 60%) for 7 days. During this period, most of the water evaporated and the containers were adjusted to the same weight by the addition of fresh distilled water. After this, all the containers contained 50 g (mL) of water. Then 100 mM NaCl solution was added in the final volume of 150 mL. Final concentration of the salt was 75 mM. The plants were then left to grow, analyzed visually and photographed at certain time-points.

### Preparation of cell membranes and two-dimensional electrophoresis

Leaves from WLSA were frozen at −80°C and then dried overnight by lyophilization at 5°C. Dried material was homogenized by bead-beating (2 × 10 s) with 2.5 mm metal beads (Mini-Beadbeater-16, BioSpec, USA). The resulting powder was mixed with a thylakoid buffer, pH 6.5, containing 25 mM MES, 10 mM MgCl_2_, 10 mM CaCl_2_, and 25% glycerol and bead-beaten (4 × 10 s). The obtained cell extract was centrifuged, after which the insoluble fraction was transferred into a new tube and solubilized with 2% dodecyl-β-maltoside for 15 min on ice. The solution was centrifuged at 30,000 × g for 10 min to remove any non-solubilized cell debris and the obtained solubilized membrane proteins were separated by electrophoresis using a 4 to 12% polyacrylamide clear native gel (Wittig et al., 2007). Individual components of protein complexes were resolved by incubating the gel strip from the first electrophoresis in 2% SDS and 1% dithiothreitol for 30 min at room temperature, after which the proteins were separated in a second dimension by SDS-electrophoresis using a denaturing 12 to 20% polyacrylamide gel containing 7 M urea (Dobáková et al., 2009). Proteins were stained with Coomassie Brilliant Blue, and individual protein components were identified by comparing the protein pattern with previously published results (Pagliano et al., 2012).

## Results

Thidiazuron [1-phenyl-3-(1,2,3-thiadiazol-5-yl)urea] (TDZ) is a synthetic cytokinin with the highest anti-senescence activity in Wheat leaf senescence assay (WLSA, this work) and effectively delays flower and leaf senescence in cut flowers and potted plants (Ferrante et al., 2002, 2003; Mutui et al., 2005).

In an attempt to discover urea-derived cytokinins with enhanced anti-senescence properties we synthesized three compounds which combine 1,2,3-thiadiazol-5-yl-urea (part of the TDZ molecule) and the *N*^6^-side chain of three natural isoprenoid-type cytokinins - *t*Z, *N*^6^-isopentenyladenine (iP) and dihydrozeatin (DHZ). These new compounds were designated TD-*t*Z, TD-iP, and TD-DHZ, corresponding to *N*-[(E)-4-hydroxy-3-methyl-but-2-enyl]-*N*′-1,2,3-thiadiazol-5-yl-urea, *N*-2-isopentenyl-*N*′-1,2,3-thiadiazol-5-yl-urea and *N*-(4-hydroxy-3-methyl-butyl)-*N*′-1,2,3-thiadiazol-5-yl-urea, respectively (structures are shown in Table 1). Their cytokinin activities were examined through three classical cytokinin bioassays [the tobacco callus assay, *Amaranthus* assay and WLSA (Holub et al., 1998)]. We found that these three 1,2,3-thiadiazol-5-yl-urea derivatives are more potent than their adenine analogs at reducing the loss of chlorophyll in detached wheat leaves (*Triticum aestivum* L.), while their cytokinin activities in the other two bioassays were the same as, or less than, the activities of the analogs (Table 2). The fact that TD-DHZ, a compound with a saturated side chain, exhibited higher activity in the WLSA than BA motivated the synthesis of a series of fifteen 1,2,3-thiadiazol-5-yl-urea derivatives with a range of aliphatic substituents (Table 3). Out of the more than five hundreds of compounds with cytokinin-like structures tested in our department, only the derivatives with unsaturated N^6^-side chains (mostly derivatives of *trans*-zeatin, BA, N^6^-phenyladenine and diphenyl urea) exhibited considerable activity in WLSA. The only exception to this rule is the compounds described in this work. Although many thidiazuron derivatives have been synthesized (e.g., Yip and Yang, 1986; Nisler et al., 2016), none of the compounds reported here have previously been described. All of the synthesized compounds were characterized by high resolution Mass Spectrometry and ^1^H NMR (Supplementary Tables S1, S2). The HPLC purity of all the compounds was always above 97%.

**Table 1:** Structures of TDZ and adenine- and 1,2,3-thiadiazol-5-yl urea-based cytokinins. *t*Z - *trans*-zeatin, iP-N^6^-(Δ^2^-isopentenyl)adenine, DHZ–dihydrozeatin.

** 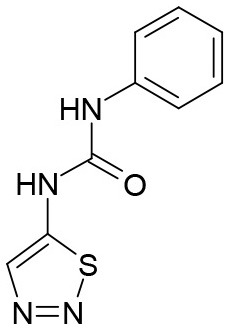 **	** 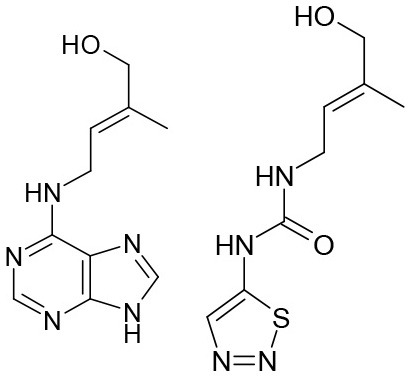 **	** 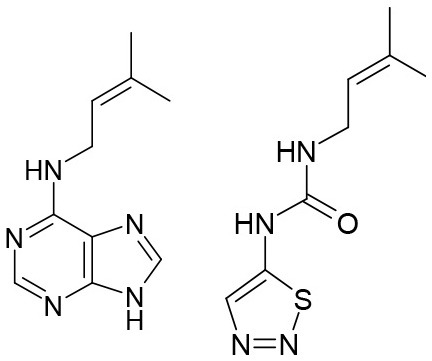 **	** 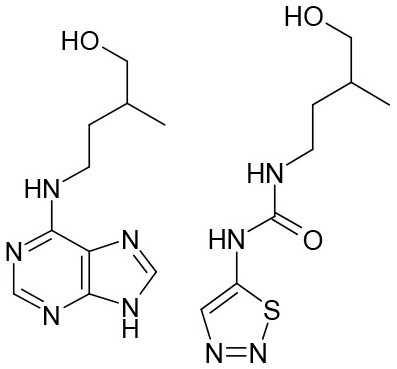 **
TDZ	*t*Z TD-*t*Z	iP TD-iP	DHZ TD-DHZ

**Table 2:** Cytokinin activities of the synthesized compounds (TD-*t*Z, TD-iP, TD-DHZ and compounds **1**-**15**) in classical cytokinin bioassays, compared with the activities of TDZ, BA, *t*Z, iP and DHZ.

**Compound**	**Senescence bioassay (IC**_**50**_, μ**M)**		**Tobacco callus bioassay (EC**_**50**_, μ**M)**		***Amaranthus*** **bioassay (EC**_**50**_, μ**M)**	
TDZ	13	(±4)	0.001	(±0.003)	0.004	(±0.0022)
BA	>100		0.060	(±0.03)	0.75	(±0.13)
*t*Z	30	(±6)	0.012	(±0.005)	0.9	(±0.4)
TD-*t*Z	13	(±2)	0.010	(±0.003)	1.5	(±0.4)
iP	>200		0.013	(±0.005)	2.0	(±0.3)
TD-iP	93	(±15)	0.017	(±0.002)	1.1	(±0.4)
DHZ	>100		0.065	(±0.021)	10	(±2.3)
TD-DHZ	56	(±10)	0.82	(±0.17)	43	(±5.5)
1	26	(±6)	15	(±3)	N.A.	
2	8.0	(±1)	7.0	(±1)	75.5	(±7.5)
**3–ASES**	**0.95**	**(**±**0.24)**	0.68	(±0.05)	4.0	(±0.8)
4	19	(±4)	2.2	(±0.06)	16.7	(±4.1)
5	3.5	(±0.6)	7.1	(±1.4)	N.A.	
6	25	(±3)	N.A.		N.A.	
7	62	(±7)	N.A.		N.A.	
8	2.5	(±0.7)	2.4	(±0.7)	16.7	(±1.9)
9	>100		70	(±4.6)	>100	
10	8.6	(±3)	3.1	(±1.1)	26.5	(±4.8)
11	83	(±17)	N.A.		>100	
12	17	(±3)	2.4		15.3	(±3.9)
13	7.1	(±1.5)	1.6		14.2	(±2.4)
14	14	(±1)	0.87	(±0.04)	10.8	(±2.5)
15	N.A.		N.A.		N.A.	

*Compounds designated by an abbreviation and ASES were tested three times in each assay while compounds **1**–**15** (except ASES) were tested only twice. The compounds were also tested in a wider concentration range to obtain their concentration-dependent activity curves. These curves were evaluated using Scion image program to obtain the IC_50_ and EC_50_ values for each compound. The parentheses show the s.d. of the mean of independent assays. (IC_50_-compound concentration at which chlorophyll degradation is inhibited by 50%; EC_50_-half the maximal effective concentration; N.A., not active). The bold values indicate IC_50_ of ASES in the wheat leaf senescence assay*.

**Table 3:** Structures of the synthesized compounds.

General structure	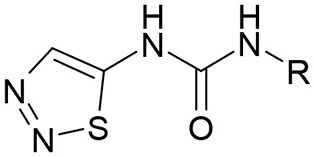		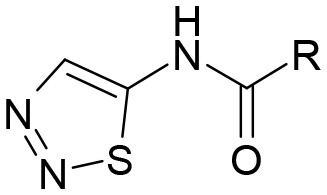

			For compounds **6** and **7**
Compound R		Compound R	
**TDZ**	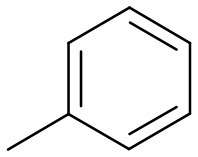	**8**	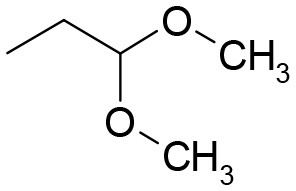
**1**	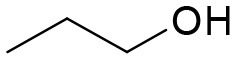	**9**	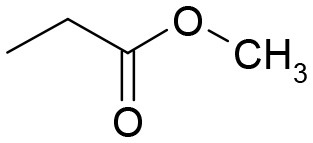
**2**	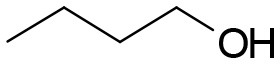	**10**	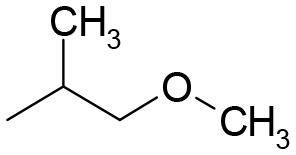
**3** (ASES)	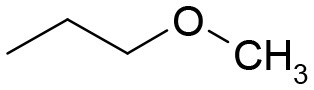	**11**	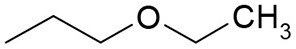
**4**	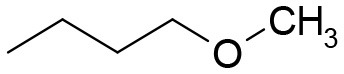	**12**	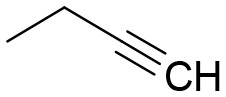
**5**	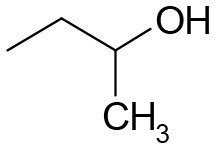	**13**	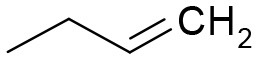
**6**	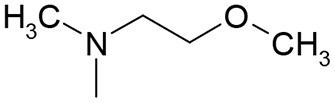	**14**	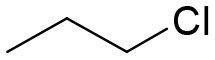
**7**	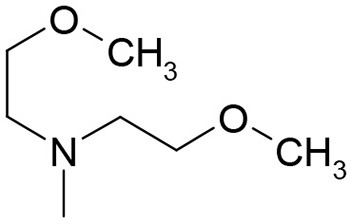	**15**	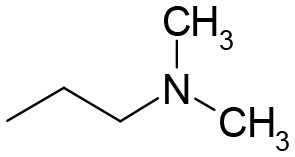

*All compounds contain the 1,2,3-thiadiazol ring and urea bridge as well as TDZ. Compounds **6** and **7** are di-alkylated at one urea nitrogen*.

### Activity in cytokinin bioassays

We used three cytokinin bioassays to investigate the complex cytokinin-related properties and structure-activity relationships of the synthesized urea derivatives. The activity of the compounds was compared with that of *N*^6^-benzyladenine (BA), *trans*-zeatine (*t*Z) and TDZ (Table 2).

**Figure 1:**
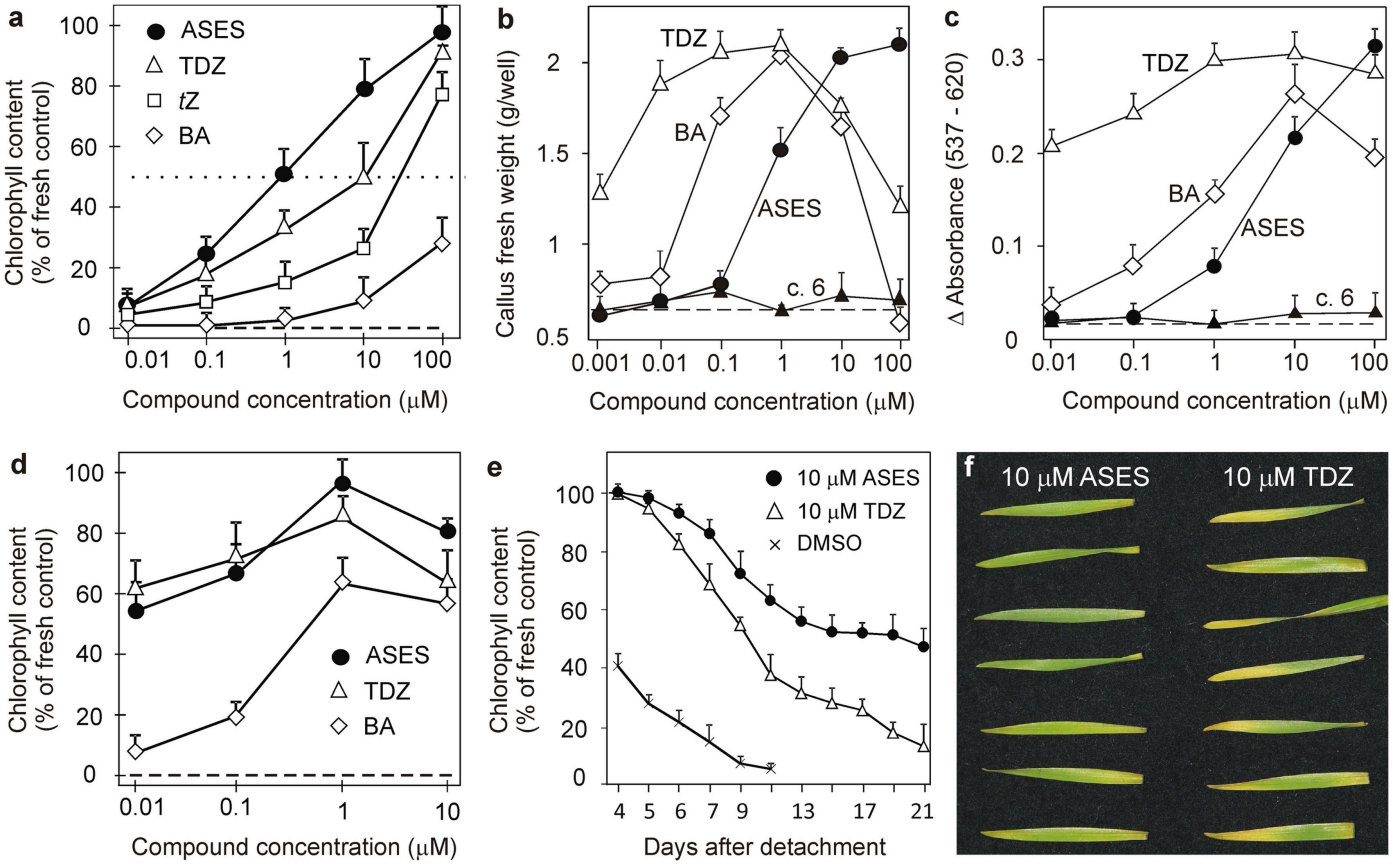
Evaluation of biological activities of selected compounds in classical cytokinin bioassays. The activity of ASES is compared with the activity of BA and TDZ. **(a)** Wheat leaf senescence assay performed in the dark (5 days). The dotted line indicates where the chlorophyll content in the leaves is 50% of that of fresh control leaves. The IC_50_ values were determined at this threshold. **(b)** Effect on growth of cytokinin-dependent tobacco callus. **(c)** Effect on dark betacyanin synthesis in *Amaranthus caudatus* cotyledon-hypocotyl explants. Graphs **a**–**c** are representative examples of compound biological activities obtained in one assay. Error bars show s.d. of the mean for five replicate determinations. c. 6 means compound **6**. **(d)** Arabidopsis leaf senescence assay performed in the dark (12 days). Error bars show s.d. of the mean for two independent assays, where each value consist of four replicates. **(e)** Time-dependent progress of chlorophyll degradation in wheat leaf senescence assay and **(f)** appearance of the leaves on the 17th day. Error bars show s.d. of the mean for four replicate determinations. In **(a,d,e)**, 100% represent chlorophyll content in fresh control leaves. Dashed lines indicate values obtained for the control treatment (DMSO control) with no added compound.

WLSA evaluates a compound's ability to retard chlorophyll degradation as a result of the stress-induced leaf senescence. In our experiments, we determined the IC_50_ value (compound concentration at which chlorophyll degradation is inhibited by 50%) for each compound (Table 2). Of the naturally-occurring cytokinins, *t*Z was the most active. However, the synthetic cytokinin TDZ showed even higher activity than *t*Z (Table 2). Furthermore, we found that 1-(2-methoxy-ethyl)-3-1,2,3-thiadiazol-5-yl-urea (compound **3** = **ASES** - **A**nti-**SE**nescence **S**ubstance) had higher activity than TDZ or *t*Z, the two most active cytokinins (Table 2, Figure 1a). ASES was approximately 13 times more effective than TDZ (Table 2). The other compounds that were more potent than TDZ and had IC_50_ values below 10 μM were: **8**< **5**< **13**< **2**< **10**. Compounds **5**, **6**, and **10** are structurally similar to ASES but methylated at different positions, and showed lower activities than ASES. Compounds bearing other electron-donating substitutions, such as compounds **1**, **4**, **6**, **12**, **13**, and **8**, were also very active in this assay, exceeding the activity of *t*Z. To generalize, the majority of the 1,2,3-thiadiazol-5-yl-ureas that include an aliphatic side chain exhibit stronger anti-senescence activity than natural cytokinins. To the best of our knowledge, ASES exhibits the strongest anti-senescent activity among all of the compounds previously described in the literature.

The tobacco callus assay measures the ability of cytokinins to promote cell division in the presence of auxin. We determined the EC_50_ values (half the maximal effective concentration) for all compounds. TDZ (EC_50_ = 1 nM) exhibited the highest activity, exceeding *t*Z activity at least tenfold (Table 2). The TD-*t*Z and TD-iP showed the same activities as their adenine analogs but, unlike classical cytokinins, did not inhibit the growth of callus at the highest concentration tested (100 μM). This indicates their lower toxicity. DHZ and BA had comparable activities. TD-DHZ was 10 times less active than DHZ. Two compounds-ASES and compound **14** showed EC_50_ values that were in the sub-micromolar range. However, the cell division stimulatory effect of ASES was more than 700 times lower than that of TDZ. Compounds **2**, **4**, **5**, **8**, **10**, **12**, and **13** had EC_50_ values between 1 and 10 μM. Compounds bearing two aliphatic substituents on N^3^ (**6**, **7**), as well as compounds **11** and **15**, were completely inactive in this assay. Data are summarized in Table 2 and the representative examples of the activity of selected compounds are shown in Figure 1b.

In the *Amaranthus* assay (Bamberger and Mayer, 1960), TDZ (EC_50_ = 4 nM) was again the most active compound, exceeding the activity of all other cytokinins at least 100-fold. TD-iP showed higher activity than iP and BA, whereas TD-*t*Z had slightly lower activity than *t*Z. None of the compounds (**1–15**) reached the activity of BA. Compounds **1**, **5**, **6**, **7**, and **15** were inactive in this assay, and the EC_50_ values of compounds **9** and **11** exceeded 100 μM. Most of the compounds had EC_50_ values greater than 10 μM, with the exception of ASES, which had an EC_50_ value of 4.0 μM. ASES induced the production of betacyanin with approximately 1,000-fold lower potency than TDZ and 5-fold lower potency than BA. Data are summarized in Table 2 and representative examples of the activity of selected compounds are shown in Figure 1c. We can conclude that ASES exhibited some cytokinin activity in the tobacco callus and *Amaranthus* assays; however, this activity was much lower than that of classical cytokinins.

We performed classical WLSA, but monitored the effects of ASES and TDZ (both at 10 μM concentration) on chlorophyll losses during a longer period of 21 days after leaf detachment. Differently treated leaves were kept separately in 1 L box. Control leaves entirely lost chlorophyll after 9 days. TDZ-treated and ASES-treated leaves contained approximately 15 and 50% of the initial chlorophyll after 21 days, respectively (Figure 1e). The appearance of the leaves on the 17th day is shown in Figure 1f. This experiment again demonstrates that ASES is more effective at inhibiting the chlorophyll loss in wheat leaves than TDZ.

We have performed the senescence assay also with *Arabidopsis thaliana* leaves, to evaluate the ASES anti-senescence properties in dicotyledonous plant. The activity of ASES was compared to those of BA and TDZ (Figure 1d). All compounds reach their maximal activity at 1 μM concentration. BA exhibited the lowest anti-senescence activity from the tested compounds, retaining by 65% more of chlorophyll than DMSO (negative control). TDZ and ASES (both 1 μM) retained by 85 and 96% more of chlorophyll than DMSO, respectively. In contrast to BA, TDZ, and ASES were highly active also at 0.1 and 0.01 μM concentration, both retaining more than 55% of the chlorophyll at 0.01 μM concentration, which makes them approximately 100 times more effective than BA (compared to the activity of BA in 1 μM). The results further demonstrate that both compounds, ASES and TDZ retained high, but similar amounts of chlorophyll in Arabidopsis leaves over the tested concentration range.

### Activation of arabidopsis receptors AHK3 and CRE1/AHK4

**Figure 2:**
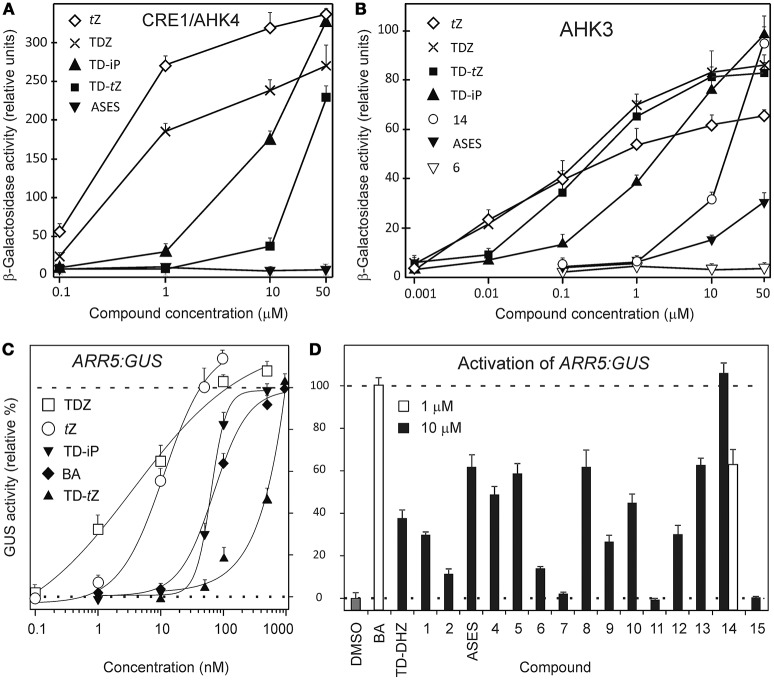
Activation of the cytokinin receptors CRE1/AHK4 **(A)** and AHK3 **(B)** by selected compounds in an *E. coli* receptor activation assay and quantitative evaluation of GUS activity in *ARR5:GUS* transgenic Arabidopsis plants **(C,D)**. *ARR5* is a cytokinin primary response gene. In all bioassays, the activities of the compounds are compared with those of *t*Z, BA (used as standards) and TDZ. Concentration-dependent curves of *ARR5:GUS* activity were constructed for the most active compounds (TD-*t*Z and TD-iP) using Scion image program **(C)**. In **(A–C)** error bars show s.d. (*n* = 3). In **(D)** the activity of compounds was compared to the activity of 1 μM BA, which was set as 100% activation (dashed line). DMSO (0.1%) was used as solvent control (dotted line).Error bars show s.d. of two parallel assays, each consisting of two replicates.

Transformed *E. coli* expressing the Arabidopsis cytokinin receptor AHK3 or CRE1/AHK4 and the cytokinin-activated reporter gene *cps::lacZ* (Suzuki et al., 2001; Yamada et al., 2001) were used to examine the ability of the synthetic compounds to activate these receptors. The EC_50_ values (Figures 2A,B) of all the synthesized compounds for both receptors were determined and compared with those of the cytokinins *t*Z and TDZ.

The EC_50_ values for *t*Z and TDZ in the CRE1/AHK4 bacterial receptor assay were 0.35 and 2.6 μM, respectively. This result is consistent with our previous study (Nisler et al., 2010), in which we determined an EC_50_ value of 0.2 μM for *t*Z. The receptor CRE1/AHK4 was activated by two urea compounds, but only at high concentrations: TD-iP (EC_50_ = 9.4 μM) < TD-*t*Z (EC_50_ = 32 μM). No other derivative from the series, including ASES, was able to activate the CRE1/AHK4 receptor (Figure 2A).

The receptor AHK3 is known to have broader ligand specificity and to be generally more sensitive than CRE1/AHK4 (Spíchal et al., 2004). This was also the case in the present study. *t*Z (EC_50_ = 66 nM) and TDZ (EC_50_ = 105 nM) were the most active compounds, followed by TD-*t*Z (EC_50_ = 190 nM) and TD-iP (EC_50_ = 1.4 μM). The AHK3 receptor was also activated by compound **14** (EC_50_ = 15 μM) and, albeit weakly, by ASES (EC_50_ > 50 μM) at the highest tested concentration. All of the other synthesized compounds were unable to activate this receptor (example for compound **6** in Figure 2B).

### Activation of the cytokinin primary response gene ARR5

All of the synthetic compounds were tested whether they could activate cytokinin responses *in planta*. We used transgenic Arabidopsis plants harboring the *ARR5:GUS* reporter gene (D'Agostino et al., 2000). *ARR5* is a primary response gene with a cytokinin-dependent promoter, which, upon activation, integrates the responses of several putative cytokinin signaling pathways.

The activity of compounds was compared with that of 1 μM BA (Figure 2D), as BA showed maximum activity at this concentration. Concentration-dependent activity curves were constructed for TDZ, *t*Z, BA, TD-iP, and TD-*t*Z to determine their EC_50_ values (Figure 2C). TDZ (EC_50_ = 4 nM) was the most active cytokinin, and showed higher activity than *t*Z (EC_50_ = 9 nM). TD-iP (EC_50_ = 65 nM) showed activity that was comparable to that of BA (EC_50_ = 76 nM), and both were more active than TD-*t*Z (EC_50_ = 0.52 μM). Of the synthetic compounds **1–15**, compound **14** was the most active, reaching 65% of BA activity at a concentration of 1 μM. Compounds **3** (ASES), **5**, **8**, and **13** were more than 10 times weaker, attaining 55–65% of BA activity at 10 μM concentrations. The activity of the remaining compounds decreased in the following order: **4** > **10** > **12** > **1** > **9** > **6** > **2**. Compounds **7**, **11**, and **15** were completely inactive. This means that, in terms of high activity, N^3^ cannot bear more than one alkyl chain (**6**, **7**), and that compounds with non-polar residue (**15**) will not activate the cytokinin signaling pathway. The results are summarized in Figure 2D. To conclude, ASES was approximately 1,000 times weaker than TDZ in activating *ARR5*.

### ASES does not inhibit the function of AtCKX2

The model plant *Arabidopsis thaliana* contains seven CKX isoforms which are involved in the regulation of endogenous cytokinin levels (Werner et al., 2003). Of these, AtCKX2 is the isoform that shows the highest activity and has been the most studied (Galuszka et al., 2007). TDZ is a known inhibitor of the CKX enzyme (Kopecný et al., 2010); hence, all the prepared compounds were tested for their AtCKX2 inhibitory activity. In contrast to TDZ, neither ASES nor any of the other synthesized substances inhibited AtCKX2 (Supplementary Figure S1). This result suggests that the observed anti-senescence effect of the tested compounds is unlikely to result from a potential elevation of endogenous cytokinin levels in plants by inhibition of the enzyme involved in their degradation.

### The effect of ASES on cytokinin levels

To exclude the possibility that ASES significantly elevates cytokinin levels in wheat we analyzed the content of individual cytokinin forms in detached leaves from the WLSA. The most relevant data are described here. The leaves were treated by DMSO (control), 10 μM TDZ or 10 μM ASES. The data are also compared to untreated leaves (fresh control) which were not exposed to dark for 5 days. The analysis showed that TDZ- and ASES-treated leaves contained a similar amount of *t*Z, which was 8.8-fold and 6.0-fold higher than in fresh control leaves, respectively. DMSO control leaves contained even 39-fold higher content of *t*Z than fresh control leaves (Table 4). The content of *t*Z-riboside was exactly the same in both TDZ- and ASES-treated leaves and, again higher (25-fold) than in fresh control leaves. *T*Z-riboside possesses the same activity in WLSA and in AHK3 receptor activation assay as *t*Z (Holub et al., 1998; Spíchal et al., 2004). In Arabidopsis, AHK3 receptor plays a dominant role in cytokinin-mediated control of leaf senescence (Kim et al., 2006). Interestingly, ASES-treated and fresh control leaves contained the same amount of *t*Z-type cytokinins, which was significantly lower than in DMSO- and TDZ-treated leaves (Table 4). Importantly, the fresh control and ASES- and TDZ-treated leaves contained the same amount of cytokinin free bases [comprising of *t*Z, *cis-*zeatin (cZ), DHZ and iP], which are the most active cytokinin forms (Spíchal et al., 2004). The total content of the free cytokinin bases in DMSO control leaves was approximately twofold higher than in the other samples. From all cytokinin forms the only cytokinin *c*Z-O-glucoside (*c*ZOG) was significantly increased in ASES-treated leaves (approximately 2.2-fold) when compared to TDZ-treated leaves. However, cytokinin glucosides are inactivated cytokinin forms (e.g., Holub et al., 1998; Veach et al., 2003). Only because of the elevated level of *c*ZOG, the total cytokinin content in ASES-treated leaves was 1.7-fold or 1.5-fold increased when compared to TDZ- or DMSO-treated leaves, respectively. These data demonstrate that ASES elevate neither the levels of active cytokinin forms nor the levels of *t*Z-type cytokinins in wheat when compared to TDZ-treated or control leaves (Table 4). Therefore, the cytokinin content analysis does not support the hypothesis that ASES causes changes in cytokinin levels that would favor to delayed senescence of ASES-treated leaves. The complete analysis data are in Supplementary Table S3.

**Table 4:** Cytokinin levels (pmol/g FW) in leaves from the wheat leaf senescence assay.

**Cytokinin**	**DMSO control**	**Fresh control**		**TDZ**			**ASES**			
*t*Z	8.3 (1.0)	0.21 (0.1)	**c**	1.8 (0.3)	**c**	**b**	1.3 (0.1)	**c**	**c**	
*t*ZR	3.0 (0.5)	0.28 (0.07)	**c**	7.1 (1.8)	**a**	**b**	7.0 (0.4)	**c**	**c**	
Total *t*Z-types	27.9 (3.2)	13.2 (2.0)	**b**	17.6 (1.5)	**b**	**a**	13.8 (0.8)	**b**		**b**
*c*Z	6.5 (1.9)	3.9 (0.9)		4.3 (0.7)			3.0 (0.6)	**a**		
*c*ZOG	228.7 (64.4)	250.7 (71.8)		207.1 (3.3)			474.7 (133.8)			**a**
Total *c*Z-types	300.8 (62)	273.2 (73)		272.3 (16)			499.1 (133)			**a**
Total bases	20.2 (0.5)	11.6 (2.3)	**b**	12.0 (0.5)	**c**		11.4 (0.3)	**c**		
Total	345.1 (64.1)	296.7 (74.2)		305.3 (16.2)			521.9 (132.6)			**a**

*Detached wheat leaves (7-day-old) were treated by 0.1% DMSO, 10 μM TDZ or 10 μM ASES and kept in the dark for 5 days prior to cytokinin analysis. Statistically significant differences, determined by an ANOVA analysis, are marked with a, b, or c corresponding to P-values of 0.05 > p > 0.01, 0.01 > p > 0.001, and p < 0.001, respectively. DMSO control vs. fresh control or treatment (black letter), fresh control vs. treatment (red letter), TDZ vs. ASES (blue letter). S.D. in brackets is for three replicates*.

### The effect of ASES on root growth in arabidopsis and wheat

**Figure 3:**
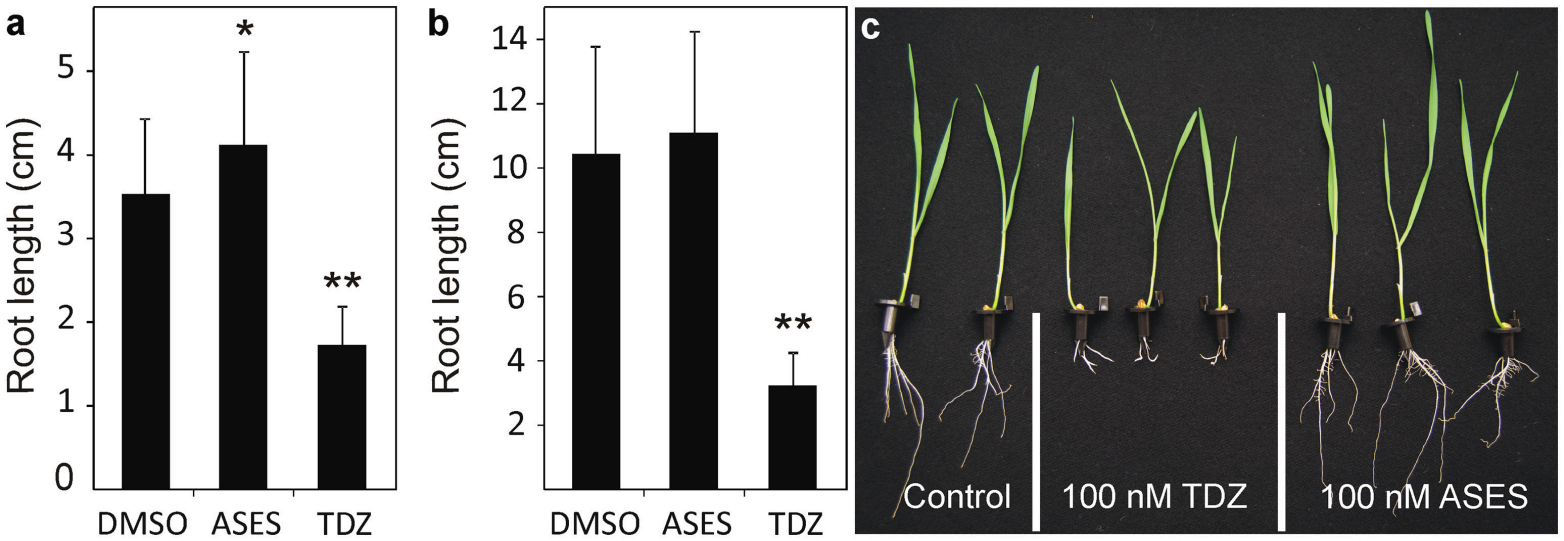
Effect of ASES and TDZ on the average length of Arabidopsis **(a)** and wheat **(b,c)** roots in a root elongation assay. The compounds were applied at a concentration of 100 nM and 0.01% DMSO was used as solvent control. Error bars represent s.d. (*n* > 40). Asterisks indicate statistically significant differences from the control treatment (Student's *t*-test, **: 0.01 > *p* > 0.001; *: 0.05 > *p* > 0.01).

To investigate whether the phenotype of ASES-treated plants resembles the phenotype of the cytokinin-treated plants Arabidopsis and wheat root elongation assays were performed. As shown in Figure 3a, treatment with 0.1 μM TDZ resulted in Arabidopsis root growth of 1.7 cm during 14 days, which was a 50% reduction when compared to the control (3.5 cm). In contrast, ASES did not impede root growth, but it stimulated root elongation in Arabidopsis (4.2 cm) compared to the control. Root branching was also unaffected by treatment with 0.1 μM ASES (data not shown). Similar results were observed with the wheat, as treatment with 0.1 μM TDZ strongly inhibited root growth (3.2 cm) in 14-day-old plants. ASES, at the same concentration, did not significantly affect the root development (11.0 cm) compared to control plants (10.4 cm, Figures 3b,c). These results clearly show that ASES does not trigger, directly nor indirectly, the cytokinin responses in wheat and Arabidopsis.

### The effect of ASES on oxidative damage in senescent leaves

**Figure 4:**
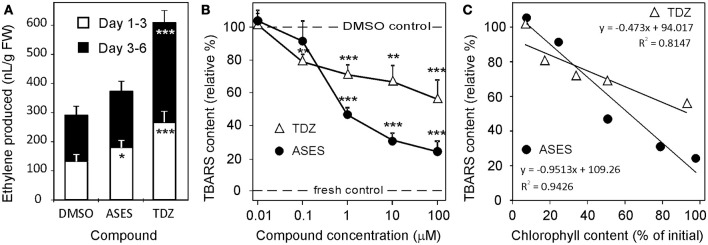
Ethylene production **(A)** of detached wheat leaves which were kept for 6 days in closed 10 mL glass vials in the dark and immersed in an aqueous solution of 0.1% DMSO (Control), 10 μM TDZ or 10 μM ASES. Error bars show s. d. (*n* = 5). **(B)** Lipid peroxidation, estimated as the relative content of TBARS, in detached wheat leaves which were kept for 5 days in the dark. Error bars show s. d. (*n* = 5). Dashed lines indicate values obtained for the control treatments. **(C)** The linear relationship between membrane lipids peroxidation and chlorophyll retention in detached wheat leaves in continuous darkness. Data used for the linear regressions are shown in Supplementary Table S4; TBARS - thiobarbituric acid-reactive substances. Asterisks indicate statistically significant difference in an ANOVA analysis (*t*-test; *, **, and *** correspond to *P*-values of 0.05 > *p* > 0.01, 0.01 > *p* > 0.001, and *p* < 0.001, respectively).

The exposure of plant tissues to stress conditions results in a dramatic elevation in the production of reactive oxygen species, which subsequently causes oxidative damage to all cellular compartments and leads to the onset of senescence (Apel and Hirt, 2004). We used the thiobarbituric acid (TBA) assay to analyze the lipid peroxidation in detached wheat leaves from WLSA and treated with TDZ or ASES. In the assay the content of TBA-reactive substances (TBARS), which are byproducts of lipid peroxidation, is determined. We found striking differences in TBARS content between treatments (Figure 4B). ASES and TDZ, both at concentrations of 100 μM, decreased lipid peroxidation to 24 and 56%, respectively, when compared to the DMSO control (100%, content of TBARS 18.4 ± 1.1 nmol/g FW). Fresh control leaves contained 9.6 ± 0.8 nmol/g FW TBARS. ASES was also more effective than TDZ at concentrations of 10 and 1 μM. A statistically significant negative linear relationship was found between chlorophyll and TBARS content in leaves treated by TDZ and ASES (Figure 4C) (Pearson's correlation coefficient *r* = −0.969 (ASES) and −0.910 (TDZ).

Levels of reactive oxygen species in plant tissues can be elevated by ethylene (Wi et al., 2010), which plays a crucial role in senescence of detached and shaded leaves (Buchanan-Wollaston et al., 2005). It has been also reported that cytokinins, even though they delay leaf senescence, increase ethylene production post-transcriptionally by increasing the activity of aminocyclopropane-1-carboxylic acid synthase 4 and 5 (Vogel et al., 1998; Woeste et al., 1999). We performed a WLSA in sealed vials to compare the amounts of ethylene produced by TDZ- and ASES-treated leaves. During the first 3 days, leaves treated with ASES and TDZ produced 1.36- and 2.02-fold more ethylene, respectively, than untreated leaves. During days 3–6, the ethylene production induced by TDZ was more than double (2.18-fold) that of the control, whereas ASES only increased ethylene production 1.22-fold (Figure 4A). To conclude, ASES-treated leaves, when compared to TDZ-treated leaves, produce less ethylene, contain less TBARS (which reflects lower oxidative damage) and contain more chlorophyll (according to Table 2). These results demonstrate that ASES is more effective in suppressing senescence-associated processes in wheat leaves than TDZ.

### The effect of ASES on salt tolerance of young wheat plants

It has been shown that the phenotypic appearance of the wheat seedlings grown under salinity stress conditions correlate well with their biochemical properties [such as the content of ascorbic acid, H_2_O_2_, MDA (TBARS in our case) and reduced glutathione, (Hasanuzzaman et al., 2011)]. Several other works also reported that there is a positive correlation between chlorophyll content, the activity of antioxidant defense system and the salt stress tolerance of various wheat cultivars (e.g., Cheng et al., 2015; Bharti et al., 2016; Jan et al., 2017; Kumar et al., 2017).

**Figure 5:**
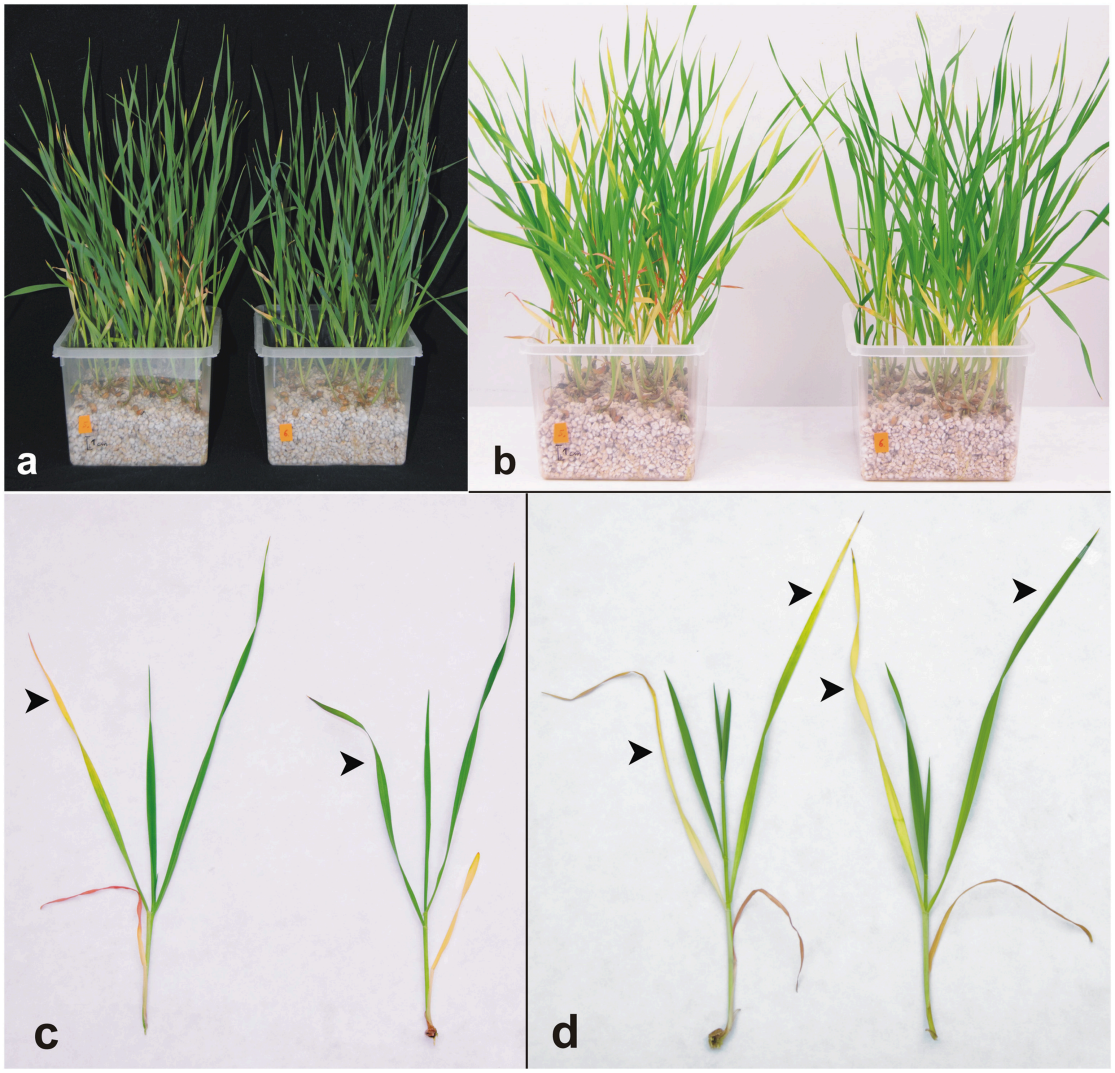
The effect of ASES on the development of wheat plants under salt stress. The plants were grown in Hoagland's solution alone (control; plants on the left) or with one application of ASES (to a final concentration of 100 nM; plants on the right) at the beginning of the experiment. Seven days after planting, the plants were exposed to 75 mM NaCl solution. Photographs were taken 20 **(a)**, 25 **(b,c)**, and 30 **(d)** days after planting. The arrows point to color differences.

We used phenotypic analysis to verify that ASES inhibits stress-induced senescence on the whole plant level. We exposed young wheat plants to salt stress, where oxidative damage plays also an important role. Control plants and plants grown in the presence of 100 nM ASES were left to grow in optimal conditions for 7 days. Then salinity stress (corresponding to 75 mM NaCl solution) was applied and the plants were analyzed visually and photographed at certain time-points. The first visible signs of senescence in the control plants occurred in the first leaf around 15th day after planting. Twenty days after planting, 81% of the control plants (55 from 68) had a dry and/or completely yellow at least one quarter of the first leaf. For comparison only 8% of the ASES-treated plants (5 from 61) exhibited the same damage (see example in Figure 5a). Twenty-five days after planting 100% of the control plants had the first leaf completely yellow and with a dry leaf tip and, the second leaves of 75% plants exhibited visible yellowing in at least one half of the leaf. At the same time, the first leaves of ASES-treated plants were only yellow (still with some chlorophyll islands) and, none of the plants had visible signs of yellowing in more than one half of the second leaf (see example in Figures 5a,b). From these results we estimate that the senescence of the individual leaves was delayed by 5–6 days in ASES-treated plants when compared to the untreated control. Senescence of the other leaves at the whole plant level was delayed to the same extent (Figures 5c,d), showing that ASES effectively enhances salt stress tolerance in wheat. Very similar results were achieved when plants were sprayed by 10 μM ASES (Supplementary Figure S2), demonstrating that ASES is absorbed by both plant roots and leaves.

### ASES prevents degradation of photosynthetic complexes

**Figure 6:**
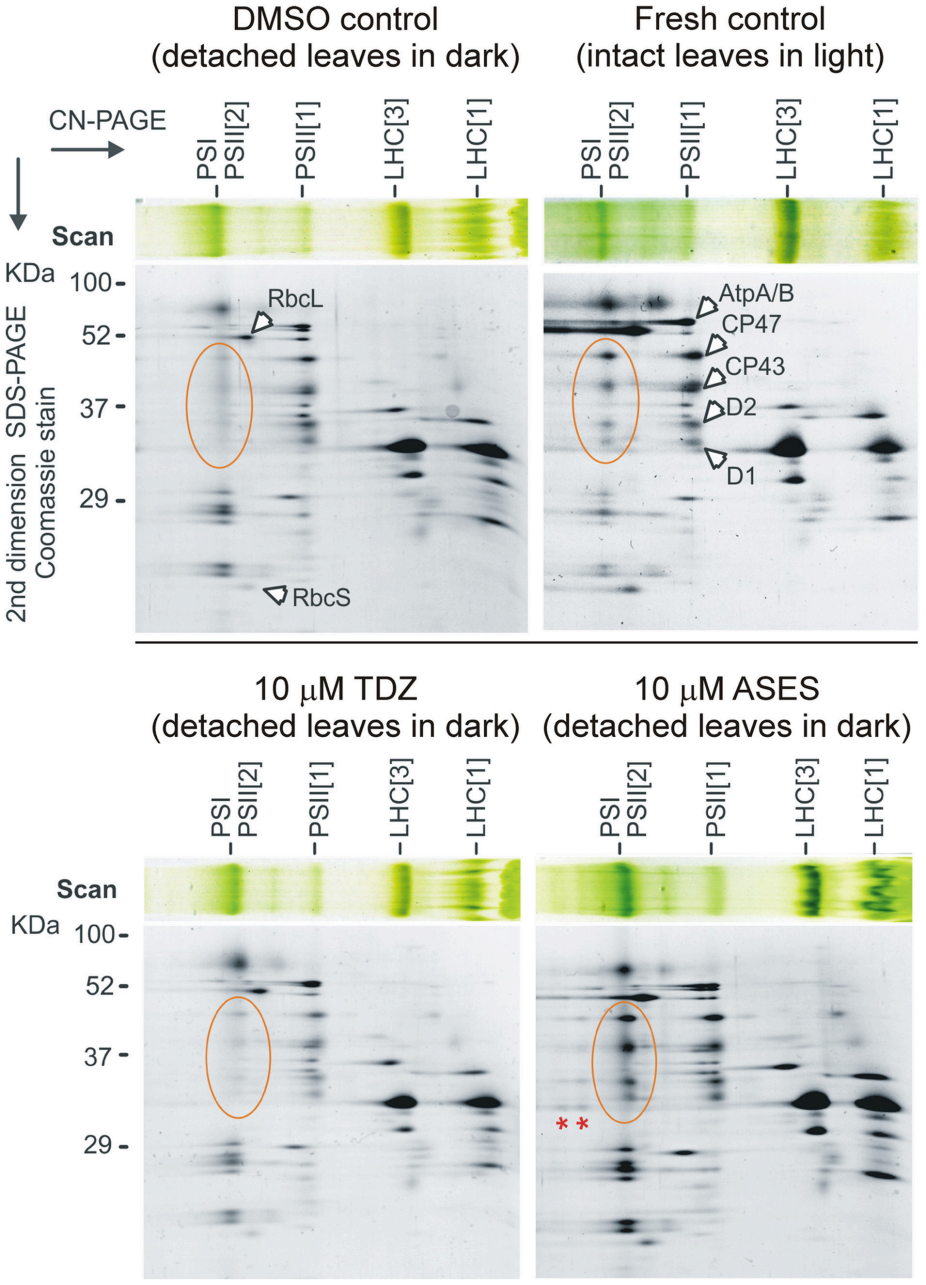
Two-dimensional clear-native/SDS electrophoresis of isolated membrane complexes. Membranes isolated from the fresh control (not detached leaves, not exposed to dark) and dark-induced senescent control (DMSO) and from the TDZ- and ASES-treated leaves (both at 10 μM concentration) were solubilized and separated by clear-native electrophoresis (CN-PAGE). The obtained protein complexes were further separated in the second dimension on a denaturing gel. Separated protein spots were identified based on previously published 2D gels of the thylakoid membrane complexes (Pagliano et al., 2012). Designation of the complexes: PSI, Photosystem I; PSII[1], PSII[2], monomer and dimer of PSII, respectively; LHC[1] and LHC[3], monomer and trimeric light-harvesting complexes II. CP47, CP43, D2, and D1 are core subunits of PSII; RbcL and RbcS are large and small Rubisco subunits; AtpA/B is α- and β-subunit of plastid ATP synthase. Orange-circled are PSII core proteins of dimeric PSII; red asterisks indicate PSII-LHCII supercomplexes.

To clarify the observed delay in senescence after ASES treatment, senescent leaves from WLSA were lyophilized, homogenized by bead-beating and the composition of chloroplast membrane proteins was analyzed in detail. We performed identical analysis on DMSO-, ASES- and TDZ-treated leaves (both at 10 μM concentration) which were exposed to injury and dark for 5 days (senescent leaves from WLSA) and compared it to the analysis of fresh control leaves (not senescent and not exposed to stress) (Figure 6).

As a first step, we measured the chlorophyll concentration in 25 mg of dried and homogenized leaf pounder of each sample. Solubilized membrane proteins prepared from the homogenized leaves (see Material and Methods section) were then loaded onto a clear-native electrophoresis gel according to a determined chlorophyll *a* concentration per dry weight. Obtained gel strips from the clear-native electrophoresis were further separated in a second dimension using SDS-electrophoresis.

The protein pattern of the DMSO control sample revealed a relatively high abundance of the Photosystem I as well as the dissociated (or free) Light-harvesting complexes II (LHCII). Although the content of membrane-bound Rubisco was quite reduced in senescent leaves, the most striking difference between fresh and DMSO controls was in the level of dimeric PSII; this complex was almost completely missing in the senescent leaves (DMSO control). The TDZ-treated leaves appeared similar to the DMSO control leaves, confirming that the effect of TDZ on the synthesis/degradation of PSII complexes is rather subtle. In contrast, the membrane complexes isolated from the ASES-treated leaves were very similar to those of the fresh control, including high levels of dimeric PSII. It is also remarkable that the ASES-treated plants exhibit even higher level of PSII-LHCII supercomplexes than we observed for fresh control; in TDZ-treated leaves these large complexes are completely lost (Figure 6). This result suggests very high stability of PSII-LHCII supercomplexes after ASES application.

## Discussion

TDZ (synthetic) and *t*Z (natural) are the most active cytokinins in their class. The WLSA showed that ASES, and several other compounds presented here, inhibit chlorophyll degradation in wheat with higher efficiency than *t*Z and TDZ. This was a surprising result, when considering that these compounds, including ASES, exhibited no or low activities in other cytokinin bioassays. We hypothesized that their anti-senescence effect is not associated with their cytokinin activity. This would mean that these compounds delay plant senescence by mechanism(s) which are not known, as most of the known mechanisms are related to the action of cytokinins (reviewed in the Introduction).

Our results support this hypothesis. Arabidopsis possesses only three cytokinin receptors–AHK2, AHK3, and CRE1/AHK4; the AHK3 receptor has been shown to be the key element in cytokinin-mediated leaf longevity (Kim et al., 2006). TDZ activates both receptors in a nanomolar range, while ASES did not activate the receptor CRE1/AHK4 at all (Figure 2A) and the receptor AHK3 was activated only very weakly (Figure 2B). However, we showed that ASES inhibited the senescence of Arabidopsis leaves comparably to TDZ.

We also excluded the possibility that ASES could increase cytokinin content in Arabidopsis by inhibiting AtCKX2 - the main enzyme of their degradation. Further, to support our hypothesis, we have analyzed the activation of transcription of the Arabidopsis cytokinin response regulator *ARR5* (D'Agostino et al., 2000). This was performed *in vivo* with transgenic Arabidopsis plants treated by the tested compounds (Figures 2C,D). The activation of all the cytokinin receptors, which do not distinguish between the endogenous and exogenous cytokinins, contributes to the activation of *ARR5* gene. In this assay ASES triggered the cytokinin response in Arabidopsis with 1,000-fold lower potency than TDZ and *t*Z. Further evidence that ASES does not exhibit cytokinin effects in Arabidopsis is given by the root elongation assay. It is a very sensitive assay in which the negative effect of TDZ on root growth is visible even at one nanomolar concentration (our unpublished results).

We assume that the same situation exists in wheat, although it is a monocotyledonous plant and its cytokinin receptors may therefore differ from those of Arabidopsis in terms of substrate specificity. However, it has been shown that the three *Zea mays* cytokinin receptors ZmHK1, ZmHK2, and ZmHK3a are homologous to the Arabidopsis receptors CRE1/AHK4, AHK3, and AHK2, respectively (Lomin et al., 2011). To strengthen the credibility of our hypothesis, we compared the content of individual cytokinin forms in TDZ- and ASES-treated leaves from WLSA. Interestigly, the content of all cytokinin forms, with only one exception, was the same or lower in ASES-treated leaves than in TDZ-treated leaves. The exception was *c*ZOG whose content was approximately twofold higher in ASES-treated leaves than in all other samples. However, cytokinin-glucosides are inactivated cytokinin forms (originated from free cytokinin bases, see e.g., Veach et al., 2003) and showed no activity in WLSA (Holub et al., 1998). Therefore, the higher content of *c*ZOG in ASES-treated leaves cannot be responsible for the ASES anti-senescence effect. Moreover, *cis*-zeatin as a free base exhibits a very low anti-senescence activity in WLSA (Gajdošová et al., 2011), having an IC_50_ of approximately 300 μM. This is 10-fold and 300-fold higher than IC_50_ of *t*Z or ASES, respectively. Therefore, even if we would assume that *c*ZOG in ASES-treated leaves originated from *c*Z, the only twofold increase of *c*Z (in ASES- versus DMSO treated leaves) could not be responsible for such a strong anti-senescence effect of ASES in WLSA. To conclude, the analysis of cytokinin levels does not support the hypothesis that ASES causes changes in cytokinin levels that would be expected to have strong anti-senescence effects. Further evidence that ASES effectively protects wheat leaves in stress conditions without triggering the cytokinin responses, was given by two *in vivo* experiments - the salt stress experiment and wheat root elongation assay. Given that the salt stress experiment only serves as a demonstration of the effect of ASES on the whole plant level, a detailed analysis of the antioxidant defense system was not performed. We believe that these data are not relevant for this article, because we expect that ASES does not primarily interact or enhance the activity of the antioxidant defense system. We also expect that the lower content of TBARS in ASES-treated leaves (when compared to both DMSO and TDZ treatment) was the result of the ASES-induced higher stability of photosynthetic protein complexes but not *vise versa*. This is supported by Tian et al. (2012) who showed that wheat stay-green mutant *tasg1* also contained less MDA than wild-type cultivar in optimal and stress conditions. Moreover, we showed that ASES-treated leaves produced the same (or higher) amount of ethylene as control leaves (DMSO-treated), but at the same time ASES-treated leaves contained significantly less TBARS. This indicates that ASES does not block the events associated with the production of ethylene in wheat leaves, which subsequetly usually leads to a higher oxidative damage (Wi et al., 2010). Indeed, this supports our hypothesis that ASES-induced inhibition of chlorophyll degradation leads to a lower oxidative damage at least in detached wheat leaves in the dark.

Regarding the root assays, in Arabidopsis, the root inhibitory activity of cytokinins is mediated through activation of the receptor CRE1/AHK4 (Riefler et al., 2006). Consistently with this, CRE1/AHK4 receptor was not activated by any of the compounds **1–15**, including ASES. This lack of effect on the plant root system is a very important characteristic of these compounds. Unlike classical cytokinins, these compounds could be used to treat seeds and seedlings. This could be pivotal to the care of seedlings and young plants that are more sensitive to stress conditions than adult or maturing plants.

It is worth mentioning that it has been previously suggested that aromatic cytokinins, especially *o-* and *m-*methoxy- and halogen-derivatives of BA, preferentially protect the degradation of the photosynthetic apparatus (Tarkowská et al., 2003; Dolezal et al., 2006). This suggestion was based on the findings that substituted aromatic cytokinins have anti-senescence activities similar to that of *t*Z, but activate Arabidopsis cytokinin receptors AHK3 and CRE1/AHK4 only weakly (Spíchal et al., 2004). In this respect we have to highlight the results achieved with compound **6** (an N^3^-methylated derivative of ASES). It showed an anti-senescence activity comparable to that of *t*Z in WLSA, but zero activity in the other cytokinin assays (Table 2, Figures 1a–c). The compound is not a cytokinin. This fact also supports our hypothesis that another mechanism by which ASES and the other compounds described herein retard chlorophyll degradation, and which differs from the cytokinin mode of action, must exist. However, we assume that ASES may inhibit leaf senescence by several mechanisms that act synergistically. We also admit that ASES weakly activated AHK3 receptor and (most probably) consequently the expression of *ARR5:GUS* gene, which both can contribute to its high *in planta* efficacy.

A part of the puzzle of how ASES delays senescence, was uncovered by the analysis of chloroplast membrane proteins in ASES- and TDZ-treated leaves. Stability of PSII-LHCII supercomplexes during senescence resembles the stay-green phenotype of Arabidopsis mutant lacking Thylakoid Formation 1 protein (Huang et al., 2013). We are therefore tempted to speculate that ASES blocks a regulatory event that triggers the degradation of PSII-LHCII supercomplexes as the first step of PSII degradation. The ASES target(s) might be enzymes required for catabolism of chlorophylls or proteins that control LHCII degradation (e.g., STAY-GREEN1 and STAY-GREEN2 in Arabidopsis). Defects in these proteins result in stable PSII levels in senescent leaves (Sakuraba et al., 2014, 2015). Alternatively, ASES might interfere with the regulation of chlorophyll biosynthesis, keeping this pathway active during senescence. In the dark-induced senescent leaves, *de novo* chlorophyll formation is very limited (Hukmani and Tripathy, 1994) but a long-term maintaining of PSII complexes also requires availability of *de novo* chlorophyll molecules (Sobotka, 2014). The stability of dimeric PSII is particularly sensitive to chlorophyll deficiency, at least in cyanobacteria (Kopecná et al., 2013). It is therefore possible that ASES maintains active *de novo* chlorophyll biosynthesis and/or inhibits chlorophyll/LHCII degradation, but the target of ASES can be also located very up-stream in the regulatory cascade (e.g., a nuclear transcription factor). This needs to be addressed in future studies.

## Conclusion

We have reported the development of compounds which are, to the best of our knowledge, currently the most efficient substances in inhibiting chlorophyll degradation in detached wheat leaves. Moreover, we demonstrate that the inhibitory mechanism is different from the mode of action of cytokinins. We further showed that, the most potent compound—ASES—also delayed senescence of Arabidopsis leaves, and again also with remarkable efficiency. We demonstrated that, *in vivo*, ASES enhanced salt tolerance in wheat in 100 nM concentration. Importantly, ASES, unlike the classical cytokinins, does not affect the development of the plant root system. This finding, together with the senescence-delaying character, could make the reported compounds relevant for the biotechnology and agricultural industries. We are also convinced that the identification of ASES molecular target(s) will shed more light on the regulation of stress and senescence processes in plants.

## Author contributions

JN designed the compounds. JN and MZ performed the synthesis of all compounds. JN performed all cytokinin bioassays. RS, JP, and BZ performed the chloroplast membrane protein analysis. ON performed cytokinin content analysis. MS and LS supervised the project. JN and RS wrote the manuscript.

### Conflict of interest statement

The authors declare that the research was conducted in the absence of any commercial or financial relationships that could be construed as a potential conflict of interest.
