# Supplementary material for: New Urea Derivatives Are Effective Anti-senescence Compounds Acting Most Likely via a Cytokinin-Independent Mechanism
Source: Front Plant Sci. 2018 Sep 11;9:1225. doi: 10.3389/fpls.2018.01225 (PMC6142817; doi:10.3389/fpls.2018.01225)

Supplementary Material

New urea derivatives are effective anti-senescence compounds acting most likely *via* a cytokinin-independent mechanism

Jaroslav Nisler[1,2,]*, Marek Zatloukal[2], Roman Sobotka[3], Jan Pilný[3], Barbora Zdvihalová[3], Ondřej Novák[1], Miroslav Strnad[1], Lukáš Spíchal[2]

*** Correspondence:** Corresponding Author: jaroslav.nisler@gmail.com

Supplementary Table S1. Accurate mass measurement of the molecular formulas of the synthesized compounds, determined using an HPLC-(ESI+)-QqTOF system (the most abundant product ion is shown in bold type). Accurate masses were calculated and used to determine the elemental composition of the analytes with fidelity of 5 ppm.

**Compound**	**Measured mass $[M+H]^+$**	**Significant product ions (m/z)**	**Molecular Formula**	**Fidelity (ppm)**
TD-*t*Z	229.0762	221, 128, **102**, 84	$C_8H_{13}N_4O_2S$	1.3
TD-iP	213.0810	157, **128**, 102, 69	$C_8H_{13}N_4OS$	0.0
TD-DHZ	231.0732	128, **102**, 103, 87	$C_8H_{14}N_4O_2S$	1.1
1	189.0458	128, **102**, 62	$C_4H_{14}N_2O_2SCl$	-3.7
2	203.0603	128, **102**, 76	$C_6H_{11}N_4O_2S$	-2.5
3	203.0594	171, 128, **102**, 76	$C_6H_{11}N_4O_2S$	-4.4
4	217.0766	185, 128, 116, 102, **90**, 84, 73, 71, 58	$C_7H_{13}N_4O_2S$	3.2
5	203.0609	128, **102**, 76	$C_6H_{11}N_4O_2S$	3.0
6	217.0758	128, 116, **90**, 88, 58	$C_7H_{13}N_4O_2S$	-0.5
7	261.1020	229, 160, **134**, 128, 102, 70, 59	$C_9H_{17}N_4O_3S$	-0.4
8	233.0710	**201**, 128, 102, 74	$C_7H_{13}N_4O_3S$	0.9
9	217.0392	128, 102, **90**, 72	$C_6H_9N_4O_3S$	-1.4
10	217.0756	128, 116, **102**, 90, 73, 58	$C_7H_{13}N_4O_2S$	-1.4
11	217.0764	171, 128, 102, **90**, 88	$C_7H_{13}N_4O_2S$	2.3
12	183.0344	128, **102**, 82	$C_6H_7N_4OS$	1.6
13	185.0490	128, **102**, 84	$C_6H_9N_4OS$	-3.8
14	207.0115	128, **102**, 80	$C_5H_8N_4OSCl$	3.9
15	216.0919	171, 128, **115**, 102, 72	$C_7H_{14}N_5OS$	2.8

Supplementary Table S2. ^{1}H NMR data of synthesized compounds. Samples were prepared by dissolving the compounds in DMSO-d_6 or $CDCl_3$. Tetramethylsilane was used as the internal standard.

**Compound**	**^{1}H NMR data**
TD-iP	(DMSO-d_6): 1.64(3H, s, CH_3), 1.67(3H, s, CH_3), 3.72(2H, t, J=5.5 Hz, CH_2), 5.2(1H, t, J=6.0 Hz, CH), 7.10(1H, t, J=5.5 Hz, NH), 8.47(1H, s, thiadiazol-ArH), 10.66(1H, s, NH)
TD-*t*Z	(DMSO-d_6): 1.57(3H, s, CH_3), 3.03(2H, m, CH_2), 3.75(2H, s, CH_2), 5.35(H, t, J=5.30 Hz, CH), 7.20(1H, t, J=5.0 Hz, NH), 8.43(1H, s, thiadiazol-ArH), 10.94(1H, s, NH).
TD-DHZ	(DMSO-d_6): 0.80(3H, d, J=6.0 Hz, CH_3), 1.15(1H, m, CH), 1.50(2H, m, CH_2), 3.15-3.22(4H, m, 2xCH_2), 4.43(1H, t, J=5.0 Hz, OH), 6.96(1H, s(br), NH), 8.42(1H, s, thiadiazol-ArH), 10.64(1H, s(br), NH).
Compound **1**	(DMSO-d_6): 3.18(2H, q, J=5.7 Hz, CH_2), 3.41(2H, q, J=4.0 Hz, CH_2), 4.75(1H, t, J=5.7 Hz, OH), 7.41(1H, t, J=4.0 Hz, NH), 8.44(1H, s, thiadiazol-ArH), 10.93(1H, s, NH)
Compound **2**	(DMSO-d_6): 1.57(2H, qui, J_1=6.9 Hz, J_2=6.3 Hz, CH_2), 3.17(2H, q, J_1=5.2 Hz, J_2=6.3 Hz, CH2), 3.41(2H, t, J=6.0 Hz, CH_2), 7.12(1H, s(br), NH), 8.41(1H, s, thiadiazol-ArH), 11.00(1H, s, NH)
Compound **3** (ASES)	(DMSO-d_6): 3.22(3H, s, CH_3), 3.28(2H, t, J=5.73 Hz, CH_2), 3.36(2H, t, J=5.2 Hz, CH_2), 7.03(1H, t, J=5.7 Hz, NH), 8.45(1H, s, thiadiazol-ArH), 10.67(1H, s, NH)
Compound **4**	($CDCl_3$-d_1): 1.88(2H, t, J=5.1 Hz, CH_2), 3.36(3H, s, CH_3), 3.52(4H, s, 2xCH_2), 6.93(1H, s(br), NH), 8.58(1H, s, thiadiazol-ArH), 10.81(1H, s(br), NH)

Compound **5**	2.95-3.01(1H, m, CH_2), 3.10-3.16(1H, m, CH_2), 1.00(3H, d, *J*=6.1 Hz, CH_3), 3.66(1H, heptet, *J*=6.7 Hz, CH), 4.78(1H, d, *J*=4.3 Hz, OH), 6.91(1H, s(br), NH), 8.46(1H, s, thiadiazol-ArH), 10.64(1H, s, NH)
Compound **6**	($CDCl_3$-d_1): 3.08(3H, s, CH_3), 3.48(3H, s, CH_3), 3.53(2H, t, *J*=4.2 Hz, CH_2), 3.66(2H, t, *J*=4.3 Hz, CH_2), 8.33 (1H, s, thiadiazol-ArH), 9.59(1H, s(br), NH)
Compound **7**	($CDCl_3$-d_1): 3.25-3.70(8H, m, 4xCH_2), 3.60(6H, s, 2xCH_3), 8.33(1H, s, thiadiazol-ArH), 9.94(1H, s, NH)
Compound **8**	($CDCl_3$-d_1): 3.36-3.39(8H, m, CH_2, 2xCH_3), 4.38(1H, t, *J*=5.0 Hz, CH), 6.21(1H, t, *J*=5.5 Hz, NH), 8.28(1H, s, thiadiazol-ArH), 10.30(1H, s(br), NH)
Compound **9**	(DMSO-d_6): (3H, s, CH_3), 3.91(2H, d, *J*=5.5 Hz, CH_2), 7.37(1H, t, *J*=5.5 Hz, NH), 8.46(1H, s, thiadiazol-ArH), 11.05(1H, s, NH)
Compound **10**	($CDCl_3$-d_1): 1.22(3H, d, *J*=6.9 Hz, CH_3), 1.80(2H, s, CH_2), 3.37(3H, s, CH_3), 3.45(1H, q, J_1=4.0 Hz, J_2=9.7 Hz, CH), 6.11(1H, s(br), NH), 8.32(1H, s, thiadiazol-ArH), 10.07(1H, s(br), NH)
Compound **11**	($CDCl_3$-d_1): 1.26(3H, t, *J*=7.1 Hz, CH_3), 3.55-3.64(6H, m, 3xCH_2), 7.12(1H, s(br), NH), 8.60(1H, s, thiadiazol-ArH), 10.82(1H, s(br), NH)
Compound **12**	($CDCl_3$-d_1): 3.75(2H, d, *J*=5.2 Hz, CH_2), 4.97(1H, d, *J*=10.3 Hz, CH_2), 5.04(1H, d, *J*=17.2 Hz, CH_2), 5.68(1H, octet, J_1=5.2 Hz, J_2=6.9 Hz, CH), 6.13(1H, s(br), NH), 8.19(1H, s, thiadiazol-ArH), 10.14(1H, s(br), NH)
Compound **13**	(DMSO-d_6): 3.90(1H, d, *J*=2.3 Hz, CH_2), 3.91(1H, d, *J*=2.3 Hz, CH_2), 7.17(1H, s(br), NH), 7.47 (1H, t, *J*=5.2 Hz, CH), 8.46(1H, s, thiadiazol-ArH), 10.89(1H, s, NH)
Compound **14**	(DMSO-d_6): 3.45(2H, q, *J*=6.3 Hz, CH_2), 3.65(2H, q, *J*=6.3 Hz, CH_2),

	7.24(1H, t, *J*=6.3 Hz, NH), 8.47(1H, s, thiadiazol-ArH), 10.87(1H, s, NH)
Compound **15**	(DMSO-d_6): 2.18(6H, s, 2xCH_3), 3.16-3.18(4H, m, 2xCH_2), 6.97(1H, s, NH), 8.43(1H, s, thiadiazol-ArH), 10.87(1H, s, NH)

Supplementary Table S3. Complete analysis of individual cytokinin forms (pmol/g FW) in detached wheat leaves from senescence assay.

	DMSO control	fresh control		TDZ			ASES			
Total CKs	**345,06 ± 64,08**	**296,73 ± 74,24**		**305,34 ± 16,21**			**521,93 ± 132,59**			*
Bases	20,21 ± 0,48	11,57 ± 2,25	**	11,97 ± 0,48	***		11,36 ± 0,25	***		
Ribosides	6,62 ± 0,26	5,79 ± 0,15	**	12,47 ± 0,89	***	***	9,16 ± 0,58	**	***	**
Nucleotides	27,84 ± 8,21	4,49 ± 1,31	**	47,40 ± 14,32		**	4,72 ± 1,23	**		**
O-glucosides	269,26 ± 70,41	264,07 ± 73,41		222,86 ± 2,50			486,39 ± 133,54			*
N-glucosides	21,12 ± 2,19	10,81 ± 1,76	**	10,65 ± 1,02	**		10,29 ± 1,67	**		

tZ	8,25 ± 1,02	0,21 ± 0,06	***	1,84 ± 0,34	***	**	1,26 ± 0,13	***	***	
tZR	3,01 ± 0,46	0,28 ± 0,07	***	7,10 ± 1,82	*	**	7,00 ± 0,44	***	***	
tZOG	2,55 ± 0,70	4,43 ± 0,73	*	1,33 ± 0,08	*	**	0,82 ± 0,05	*	**	**
tZROG	0,064 ± 0,009	0,04 ± 0,01		0,12 ± 0,03	*	*	0,076 ± 0,021			
tZ7G	0,024 ± 0,005	0,003 ± 0,001	**	0,014 ± 0,001	*	***	0,016 ± 0,001	*	***	
tZ9G	13,96 ± 1,18	8,20 ± 1,29	**	7,22 ± 0,96	**		4,60 ± 0,27	***	**	**
tZR5'MP	*<LOD*	*<LOD*		*<LOD*			*<LOD*			
**Total *tZ*-types**	**27,86 ± 3,15**	**13,16 ± 1,99**	**	**17,62 ± 1,51**	**	*	**13,78 ± 0,75**	**		**
cZ	6,53 ± 1,87	3,88 ± 0,88		4,25 ± 0,68			3,02 ± 0,62	*		
cZR	3,24 ± 0,43	4,57 ± 0,18	**	4,55 ± 1,38			1,93 ± 0,24	**	***	*
cZOG	228,69 ± 64,40	250,73 ± 71,81		207,04 ± 3,27			**474,67** ± 133,76	*		*

cZROG	37,90 ± 5,52	8,79 ± 0,92	***	14,31 ± 0,92	**	**	10,79 ± 0,64	**	*	**
cZ7G	*<LOD*	*<LOD*		*<LOD*			*<LOD*			
cZ9G	4,55 ± 0,72	1,89 ± 0,45	**	2,47 ± 0,46	*		5,45 ± 1,57		*	*
cZR5'MP	19,86 ± 6,15	3,30 ± 1,00	*	39,70 ± 12,08		**	3,27 ± 0,87	**		**
**Total *cZ*-types**	**300,77 ± 62,10**	**273,15 ± 73,33**		**272,33 ± 16,08**			**499,14 ± 132,80**			*
DHZ	0,093 ± 0,015	*<LOD*		0,041 ± 0,002	*		*<LOD*			
DHZR	0,072 ± 0,020	0,089 ± 0,025		0,099 ± 0,020			0,100 ± 0,014			
DHZOG	0,067 ± 0,015	0,080 ± 0,008		0,053 ± 0,004		**	0,040 ± 0,003	*	**	*
DHZROG	*<LOD*	*<LOD*		*<LOD*			*<LOD*			
DHZ7G	0,164 ± 0,015	0,127 ± 0,012	*	0,175 ± 0,022		*	0,067 ± 0,006	***	**	**
DHZ9G	0,030 ± 0,007	0,016 ± 0,004	*	0,012 ± 0,003	*		0,027 ± 0,007			*
DHZR5'MP	*<LOD*	*<LOD*		*<LOD*			*<LOD*			
**Total DHZ-types**	**0,43 ± 0,01**	**0,31 ± 0,03**	**	**0,37 ± 0,02**	*		**0,23 ± 0,01**	***	*	***
iP	5,35 ± 0,92	7,48 ± 1,42		5,85 ± 0,92			7,08 ± 0,31	*		
iPR	0,30 ± 0,10	0,85 ± 0,07	**	0,72 ± 0,24	*		0,12 ± 0,03		***	**
iP7G	0,020 ± 0,003	0,010 ± 0,002	*	0,025 ± 0,004		**	0,020 ± 0,001		**	
iP9G	2,37 ± 0,31	0,57 ± 0,03	***	0,73 ± 0,12	**		0,11 ± 0,02	***	***	***
iPR5'MP	7,98 ± 2,08	1,19 ± 0,31	**	7,70 ± 2,25		**	1,45 ± 0,37	**		**
**Total iP-type**	**16,01 ± 1,17**	**10,10 ± 1,24**	**	**15,02 ± 1,54**		*	**8,79 ± 0,12**	***		**

Asterisks indicate statistically significant difference in an ANOVA analysis (t-test; *, **, and *** correspond to P-values of $0.05 > p > 0.01$, $0.01 > p > 0.001$, and $p < 0.001$, respectively). *<LOD* means under limit of detection, **DMSO control *vs* fresh control or treatment (black asterisks), fresh control *vs* treatment, TDZ *vs* AS**

Supplementary Table S4. Lipid peroxidation, estimated as the relative content of TBARS in the detached leaves of winter wheat treated with ASES and TDZ (concentration 0.01 - 100 µM). Values represent the means (± s.d., n = 5). DMSO control means the detached leaves incubated with control solvent (0.1% DMSO) 5 days in the dark. Fresh control means not detached leaves not exposed to dark. Chlorophyll retention is expressed as the percentage of initial chlorophyll content in the fresh control leaves. The linear relationship between peroxidation of membrane lipids and chlorophyll retention in detached wheat leaves in the continuous dark was assessed with Pearson's correlation.

ASES			TDZ		
**Compound concentration (µM)**	**TBARS content (relative %)**	**Chlorophyll content (% of the fresh control)**	**Compound concentration (µM)**	**TBARS content (relative %)**	**Chlorophyll content (% of the fresh control)**
**DMSO control**	100	0	**DMSO control**	100	0
**0.01**	105 (±5)	8 (±4)	**0.01**	101 (±5)	8 (±2)
**0.1**	91 (±10)	25 (±7)	**0.1**	80 (±3)	18 (±5)
**1**	47 (±4)	51 (±8)	**1**	71 (±6)	34 (±7)
**10**	31 (±5)	79 (±8)	**10**	68 (±8)	50 (±11)
**100**	24 (±6)	98 (±7)	**100**	55 (±10)	91 (±2)
**Fresh control**	0	100	**Fresh control**	0	100

Supplementary figure S1. Inhibitory strength of TDZ, ASES and compound **8** against AtCKX2. CKX activity was measured by PMS/MTT kinetic assay (Frébort *et al.*, 2002). Error bars show s.d. of three replicates. Activity of the other compounds 1-15 was similar to the ASES activity in AtCKX2 inhibition assay.

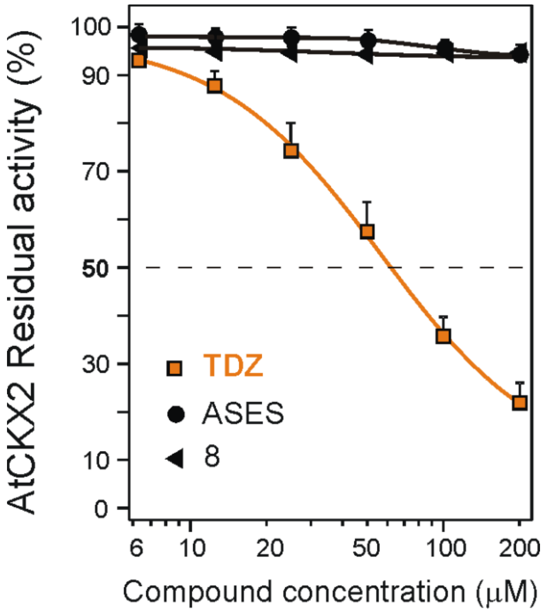

Supplementary figure S2. The effect of ASES on the development of wheat plants under salt stress. Plants were grown in Hoagland´s solution and seven days after planting the plants were treated with 75 mM NaCl solution and sprayed by 0.01% silwet (control) or 10 μM solution of ASES (solution in water + 0.01% silwet). Photographs were taken 21 days after planting.

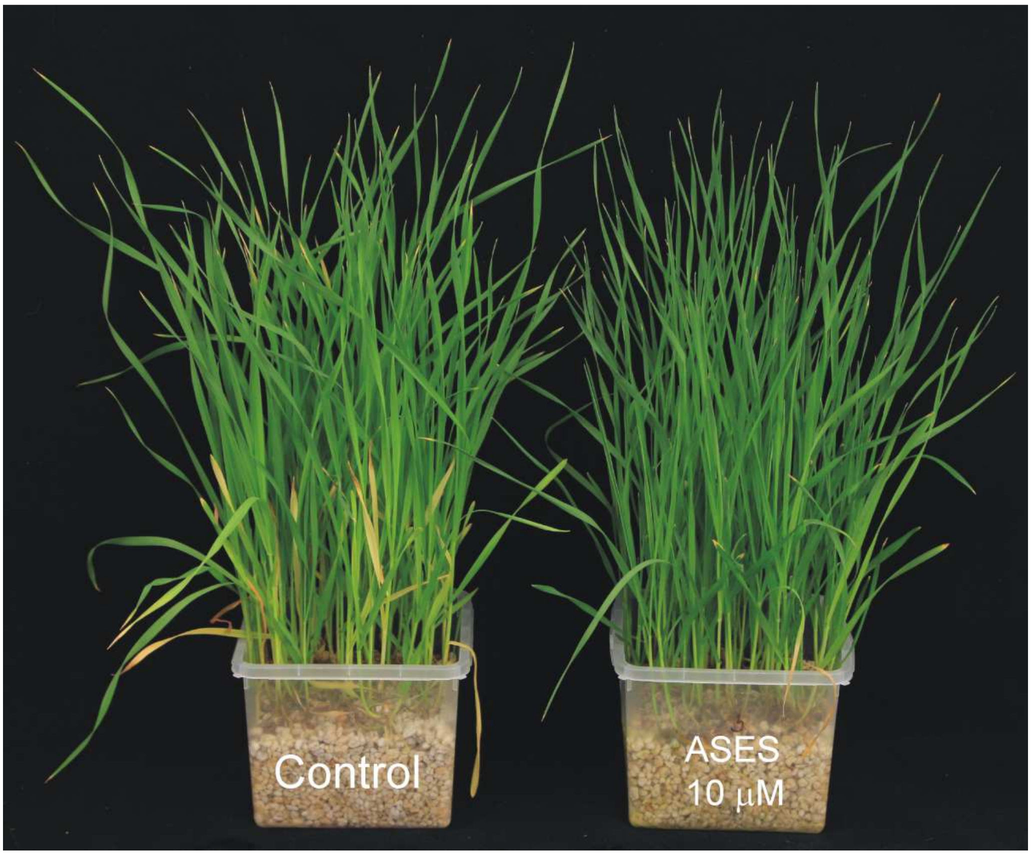

Supplement: Supplementary file 1 [file Table_1.pdf]
